# First-year treatment response predicts the following 5-year disease course in patients with relapsing-remitting multiple sclerosis

**DOI:** 10.1016/j.neurot.2025.e00552

**Published:** 2025-02-17

**Authors:** Simona Toscano, Tim Spelman, Serkan Ozakbas, Raed Alroughani, Clara G. Chisari, Salvatore Lo Fermo, Alexandre Prat, Marc Girard, Pierre Duquette, Guillermo Izquierdo, Sara Eichau, Pierre Grammond, Cavit Boz, Tomas Kalincik, Yolanda Blanco, Katherine Buzzard, Olga Skibina, Maria Jose Sa, Anneke van der Walt, Helmut Butzkueven, Murat Terzi, Oliver Gerlach, Francois Grand'Maison, Matteo Foschi, Andrea Surcinelli, Michael Barnett, Alessandra Lugaresi, Marco Onofrj, Bassem Yamout, Samia J. Khoury, Julie Prevost, Jeannette Lechner-Scott, Davide Maimone, Maria Pia Amato, Daniele Spitaleri, Vincent Van Pesch, Richard Macdonell, Elisabetta Cartechini, Koen de Gans, Mark Slee, Tamara Castillo-Triviño, Aysun Soysal, Jose Luis Sanchez-Menoyo, Guy Laureys, Liesbeth Van Hijfte, Pamela McCombe, Ayse Altintas, Bianca Weinstock-Guttman, Eduardo Aguera-Morales, Masoud Etemadifar, Cristina Ramo-Tello, Nevin John, Recai Turkoglu, Suzanne Hodgkinson, Sarah Besora, Bart Van Wijmeersch, Ricardo Fernandez-Bolaños, Francesco Patti, the MSBase Study Group

**Affiliations:** a Department of Biomedical and Biotechnological Sciences, University of Catania, Via Santa Sofia 78, 95123 Catania, Italy; b Multiple Sclerosis Unit, University-Hospital G. Rodolico - San Marco, Catania, Italy; c MSBase Foundation, VIC, Melbourne, Australia; d Department of Clinical Neuroscience, Karolinska Institute, Stockholm, Sweden; e Dokuz Eylul University, Konak, Izmir, Turkey; f Division of Neurology, Department of Medicine, Amiri Hospital, Sharq 73767, Kuwait; g Department of Medical and Surgical Sciences and Advanced Technologies, GF Ingrassia, Catania 95123, Italy; h CHUM MS Center and Universite de Montreal, Montreal H2L 4M1, Canada; i Hospital Universitario Virgen Macarena, Sevilla 41009, Spain; j CISSS Chaudière-Appalache, Levis G6X 0A1, Canada; k KTU Medical Faculty Farabi Hospital, Trabzon 61080, Turkey; l CORe, Department of Medicine, The University of Melbourne, Melbourne 3050, Australia; m Neuroimmunology Centre, Department of Neurology, Royal Melbourne Hospital, Melbourne 3050, Australia; n Center of Neuroimmunology, Service of Neurology, Hospital Clinic de Barcelona, Barcelona, Spain; o Department of Neurology, Box Hill Hospital, Melbourne 3128, Australia; p Department of Neurology, Centro Hospitalar Universitario de Sao Joao, Porto 4200-319, Portugal; q Faculty of Health Sciences, University Fernando Pessoa, Porto, Portugal; r Department of Neurology, The Alfred Hospital, Melbourne 3000, Australia; s Medical Faculty, 19 Mayis University, Samsun 55160, Turkey; t Academic MS Center Zuyd, Department of Neurology, Zuyderland Medical Center, Sittard-Geleen 5500, the Netherlands; u School for Mental Health and Neuroscience, Department of Neurology, Maastricht University Medical Center, Maastricht 6131 BK, the Netherlands; v Neuro Rive-Sud, Quebec J4V 2J2, Canada; w Department of Neuroscience, Multiple Sclerosis Center, Neurology Unit, S. Maria delle Croci Hospital, AUSL Romagna, Ravenna, Italy; x Department of Biotechnological and Applied Clinical Sciences, University of L'Aquila, L'Aquila, Italy; y Brain and Mind Centre, Sydney 2050, Australia; z IRCCS Istituto delle Scienze Neurologiche di Bologna, Bologna, Italy; aa Department of Biomedical and Neuromotor Science, University of Bologna, Bologna, Italy; ab Department of Neuroscience, Imaging, and Clinical Sciences, University G. D'Annunzio, Chieti 66013, Italy; ac Nehme and Therese Tohme Multiple Sclerosis Center, American University of Beirut Medical Center, Beirut 1107 2020, Lebanon; ad CSSS Saint-Jérôme, Saint-Jerome J7Z 5T3, Canada; ae School of Medicine and Public Health, University Newcastle, Newcastle 2305, Australia; af Centro Sclerosi Multipla, Garibaldi Hospital, Catania 95124, Italy; ag Department NEUROFARBA, University of Florence, Florence 50134, Italy; ah IRCCS Fondazione Don Carlo Gnocchi, Florence, Italy; ai Azienda Ospedaliera di Rilievo Nazionale San Giuseppe Moscati Avellino, Avellino 83100, Italy; aj Department of Neurology, Cliniques Universitaires Saint-Luc, Université Catholique de Louvain (UCLouvain), Brussels, Belgium; ak Austin Health, Melbourne 3084, Australia; al AST 3 Macerata, Marche, Italy; am Department of Neurology, Groene Hart Ziekenhuis, Gouda, Zuid-Holland, the Netherlands; an Flinders University, Adelaide 5042, Australia; ao Hospital Universitario Donostia and IIS Biodonostia, San Sebastián, Spain; ap Bakirkoy Education and Research Hospital for Psychiatric and Neurological Diseases, Istanbul 34147, Turkey; aq Department of Neurology, Galdakao-Usansolo University Hospital, Osakidetza-Basque Health Service, Biocruces, Spain; ar Department of Neurology, Ghent Universitary Hospital, Ghent 9000, Belgium; as Royal Brisbane and Women's Hospital, University of Queensland, Brisbane 4000, Australia; at Department of Neurology, School of Medicine, Koc University, Koc University Research Center for Translational Medicine (KUTTAM), Istanbul 34450, Turkey; au Department of Neurology, Buffalo General Medical Center, Buffalo 14202, United States; av University Hospital Reina Sofia, Cordoba 14004, Spain; aw Department of Neurosurgery, School of Medicine, Isfahan University of Medical Sciences, Isfahan, Iran; ax Hospital Germans Trias i Pujol, Badalona 08916, Spain; ay Department of Medicine, School of Clinical Sciences, Monash University, Clayton, Australia; az Department of Neurology, Monash Health, Clayton, Australia; ba Haydarpasa Numune Training and Research Hospital, Istanbul 34668, Turkey; bb Liverpool Hospital, Sydney 2170, Australia; bc Hospital Universitari Mútua de Terrassa, Barcelona, Spain; bd Universitair MS Centrum, Hasselt University, Hasselt-Pelt, Belgium; be Rehabilitation & MS Centre, Pelt, Belgium; bf Department of Neurology, Hospital Universitario Virgen de Valme, Spain

**Keywords:** Multiple sclerosis, Disease-modifying treatment, Prognosis, Nomogram, High-efficacy drugs

## Abstract

Predicting long-term prognosis and choosing the appropriate therapeutic approach in patients with Multiple Sclerosis (MS) at the time of diagnosis is crucial in view of a personalized medicine. We investigated the impact of early therapeutic response on the 5-year prognosis of patients with relapsing-remitting MS (RRMS). We recruited patients from MSBase Registry covering the period between 1996 and 2022. All patients were diagnosed with RRMS and actively followed-up for at least 5 years to explore the following outcomes: clinical relapses, confirmed disability worsening (CDW) and improvement (CDI), EDSS 3.0, EDSS 6.0, conversion to secondary progressive MS (SPMS), new MRI lesions, Progression Independent of Relapse Activity (PIRA). Predictors included demographic, clinical and radiological data, and sub-optimal response (SR) within the first year of treatment. Female sex (HR 1.27; 95 ​% CI 1.16–1.40) and EDSS at baseline (HR 1.19; 95 ​% CI 1.15–1.24) were independent risk factors for the occurrence of relapses during the first 5 years after diagnosis, while high-efficacy treatment (HR 0.78; 95 ​% CI 0.67–0.91) and age at diagnosis (HR 0.83; 95 ​% CI 0.79–0.86) significantly reduced the risk. SR predicted clinical relapses (HR ​= ​3.84; 95 ​% CI 3.51–4.19), CDW (HR ​= ​1.74; 95 ​% CI 1.56–1.93), EDSS 3.0 (HR ​= ​3.01; 95 ​% CI 2.58–3.51), EDSS 6.0 (HR ​= ​1.77; 95 ​% CI 1.43–2.20) and new brain (HR ​= ​2.33; 95 ​% CI 2.04–2.66) and spinal (HR 1.65; 95 ​% CI 1.29–2.09) MRI lesions. This study highlights the importance of selecting the appropriate DMT for each patient soon after MS diagnosis, also providing clinicians with a practical tool able to calculate personalized risk estimates for different outcomes.

## Introduction

Multiple Sclerosis (MS) is a chronic immune-mediated disease of the central nervous system characterized by high complexity and extreme heterogeneity in terms of clinical presentation and course. The relapsing-remitting phenotype (RRMS), that accounts for 80–85 ​% of cases, is associated with both demyelination and neurodegeneration since its early phases [1] and the accumulation of disability may occur at any stage of the disease, associated with the occurrence of relapses (relapse-associated worsening, RAW) or in the absence of relapses (progression independent of relapse activity, PIRA) [2]. While RAW predominates in the early phases of the disease and mostly in RRMS and pediatric MS, PIRA seems to affect disability worsening in all phenotypes of MS and can start at different points during the disease course, even precociously [3,4]. Nevertheless, RRMS course can be extremely variable and profoundly affected by the introduction of highly effective disease-modifying treatments (HET). In this context, a prognostic stratification since disease onset is not simple and a lot is yet to be understood about the long-term disease course and the timing of transition into a secondary progressive phenotype (SPMS).

A minority of patients, ranging between 3.4 and 14.0 ​% of the whole MS population, exhibit a “malignant” or “aggressive” disease course and several attempts have been made to reach their early identification. This condition is often recognized in retrospect in patients who achieve a score of 6.0 ​at the Expanded Disability Status Scale (EDSS) within 5 years from the onset [[5], [6], [7]] or by the age of 40 years [8], or in those who turned to SPMS phenotype within 3 years from the onset [8]. Alternatively, an aggressive course has also been defined as the occurrence within the first year after onset of at least two gadolinium-enhancing lesions at brain MRI, together with at least two clinical relapses, or even one relapse if resulting in sustained EDSS score of 3.0 [9].

A worse prognosis has been attributed to some demographic features, including male sex, older age at symptom onset, Afro-Americans and Hispanic ethnicity [10]. A higher relapse rate and shorter intervals between relapses, often with subsequent incomplete recovery, have been identified as additional risk factors. Further, PPMS phenotype and the presence of spinal cord and brainstem lesions at MRI at clinical presentation are often predictors of poor clinical outcome [5]. In a two-stage model for disability progression in MS [11], gender, age at onset, the occurrence of relapses during the first 2 years after onset and an incomplete recovery after relapses were found to be predictive factors only for the achievement of EDSS 3.0. According to the model, the subsequent phase and reaching an EDSS 6.0 were independent in terms of duration (median 6–9 years) from the time needed to reach an EDSS 3.0.

In this context, we collected clinical and radiological data of a large population of patients with RRMS, actively followed-up at different MS Centers, in order to investigate the impact of both the first disease-modifying treatment (DMT) choice and the treatment response in the first year after diagnosis on the 5-years prognosis. As a secondary aim, prognostic nomograms were built to predict the disease course at 5 years based on early clinical markers.

## Materials and methods

### Study population

In this multicenter retrospective study, we collected demographic and clinical data of patients with RRMS covering the period 1996–2022 from MSBase, a large international Registry recording routine clinical data inserted in iMed© from MS Centers in over 30 countries worldwide. Inclusion criteria were: a diagnosis of RRMS based on the existing McDonald's criteria according to epoch and country, a diagnostic delay ≤12 months, start of DMT within 12 months from diagnosis, availability of demographic, clinical and radiological data within 12 months from diagnosis and of clinical data for at least 5 years after diagnosis (Table 1). Post-baseline follow-up was defined as the time from baseline to the last visit recorded in the registry per patient.

**Table 1:** Inclusion criteria of the study population.

	N.
All patients in MSBase from recruiting centers	83,978
RRMS	68,470
Time between onset and diagnosis ≤12 months	30,943
First DMT started within 12-months of diagnosis	15,145
Minimum 5 years post diagnosis registry follow-up	7955
Baseline clinical and MRI data recorded within 12 months from diagnosis	3797

MS: Multiple Sclerosis; RRMS: Relapsing-remitting MS; DMT: disease-modifying treatment; MRI: magnetic resonance imaging.

Age at onset and sex were considered as demographics. Clinical variables included EDSS, pyramidal Functional System (FS) scores, number of relapses. Radiological data included the number of lesions counted in T2-weighted and T1-weighted post-gadolinium (Gd+) scans in brain and spinal MRI, performed by patients as for clinical routine. Treatment with DMT was reported for all patients. Particularly, interferon, glatiramer acetate, dimethyl fumarate, teriflunomide were considered as mild-to-moderate-efficacy DMT (MET), while cladribine, natalizumab, ocrelizumab, alemtuzumab, fingolimod and mitoxantrone were considered as HET. Data were extracted from a computerized database, iMed© (Merck Serono SA; Geneva), which contains clinical information inserted in real-time during outpatient visits.

### Outcomes and definitions

Primary outcomes were defined over a period of 5 years from the time of diagnosis (Table S1). Time to first relapse, confirmed worsening, conversion to SPMS and time to first PIRA were analyzed as the primary study endpoints. Time to disability improvement, milestone EDSS, and new lesions on brain MRI were analyzed as exploratory outcomes only.

Baseline was defined as the date of MS diagnosis. Diagnosis year was split into epochs as follows: pre-2000, 2000–2004, 2005–2009, 2010–2014 and 2015 onwards.

Predictor variables included demographic (age at diagnosis, sex), clinical (disease duration from onset, EDSS and pyramidal FS at baseline) and radiological data (number of T2 brain lesions, ≥1 spinal lesion, ≥1 gadolinium-enhancing brain lesions). EDSS at baseline was considered as the EDSS score recorded within 1–3 months from the last relapse occurred. Additionally, we considered as a predictor the suboptimal response after 1-year treatment with a DMT (SR), defined by the contextual occurrence of ≥1 gadolinium-enhanced lesions at brain or spine Magnetic Resonance Imaging (MRI) scans, or ​≥ ​1 relapse.

### Statistical analysis

Categorical variables were summarized using frequency and percentage. Continuous variables were summarized using mean and standard deviation (SD) or median and interquartile range (IQR) as appropriate. The identification of demographic, clinical and investigational correlates of five-year clinical outcomes were undertaken using a multilevel mixed effects parametric survival model presuming an underlying Weibull distribution. Age, sex, EDSS, time since onset, MRI lesions and SR were defined as fixed effects, whilst country and diagnosis epoch were included in the model as random effects. In order to adjust for inter-clinic heterogeneity, clinic effects were also included as random effects in the model. Hazard Ratio (HR) and 95 ​% confidence interval (CI) were provided for all variables explored and for each outcome. Subgroup analyses limited to patients on oral MET (including teriflunomide and dimethyl fumarate) were conducted for all outcomes.

Independent prognostic correlates of five-year outcome identified in the multivariable parametric survival modeling were then used to derive the prognostic nomograms using the method described by Kattan et al. [12,13], using the nomogram function of the RMS package in R [14]. Candidate multivariable models were assessed for collinearity and potential interactions between concurrent nomogram predictors. Quadratic transformations were incorporated into the models to test for the linearity of association between candidate explanatory variables and the clinical endpoints. The Akaike and Bayesian Information criteria were used to assess relative goodness of fit between multiple, competing multivariable model solutions prior to the selection of the final model for the development of the final prognostic nomogram. Internal validation of each nomogram was conducted via derivation of concordance indices and evaluation of nomogram calibration. Calibration was conducted by taking 500 bootstrapped resamples. Clinical outcome probability (as predicted by the nomogram) and the mean scores of these probability groups were then compared to the empirically observed non-response estimates on a calibration curve. No correction or imputation of missing data was undertaken. All analyses were conducted in R version 4.0.5 (R Foundation for Statistical Computing) and Stata version 16.1 (StataCorp, College Station, Texas).

## Results

### Study population

From a total of 83,978 patients recorded in the Registry from participating centers, 3797 subjects met the inclusion criteria and were enrolled in the study. Of those, 2682 (70.9 ​%) were female, and the mean age at onset was 32.15 ​± ​9.79 years. The characteristics of the study population are reported in Table 2. The mean (SD) annualized number of MRI scans per patient was 1.30 (0.85) scans per year.

**Table 2:** Baseline characteristics of the study population.

			Cohort with a legitimate baseline EDSS and MRIa
Factor	Category	n ​= ​7955	n ​= ​3797
**Age at baseline (years) - mean (SD)**		31.43 (9.79)	32.15 (9.79)
**Sex - n (%)**	Female	5652 (71.1)	2682 (29.3)
	Male	2302 (28.9)	1114 (29.3)
	Not recorded	1 (0.0)	1 (0.0)
**Months since first symptoms - mean (SD)**		4.20 (3.55)	4.27 (3.35)
**Diagnosis year - n (%)**	Pre-2000	501 (6.3)	91 (2.4)
	2000–2004	1440 (18.1)	444 (11.7)
	2005–2009	2426 (30.5)	1086 (28.6)
	2010–2014	2671 (33.6)	1503 (39.6)
	2015 onwards	917 (11.5)	673 (17.7)
**Country - n (%)**	Australia	1632 (20.5)	694 (18.3)
	Turkey	1439 (18.1)	636 (16.8)
	Italy	867 (10.9)	612 (16.1)
	Canada	676 (8.5)	479 (12.6)
	Spain	666 (8.4)	454 (12.0)
	Kuwait	453 (5.7)	182 (4.8)
	Belgium	285 (3.6)	130 (3.4)
	Iran	266 (3.3)	34 (0.9)
	Netherlands	257 (3.2)	137 (3.6)
	Portugal	157 (2.0)	107 (2.8)
	Lebanon	156 (2.0)	72 (1.9)
	United States	153 (1.9)	35 (0.9)
	Switzerland	141 (1.8)	14 (0.4)
	Egypt	92 (1.2)	1 (0.0)
	Argentina	86 (1.1)	35 (0.9)
	United Kingdom	73 (0.9)	18 (0.5)
	Tunisia	67 (0.8)	19 (0.5)
	Ireland	61 (0.8)	1 (0.0)
	Croatia	54 (0.7)	0 (0.0)
	Brazil	51 (0.6)	40 (1.1)
	UAE	47 (0.6)	0 (0.0)
	Oman	45 (0.6)	18 (0.5)
	Czechia	36 (0.5)	21 (0.6)
	Denmark	32 (0.4)	9 (0.2)
	Hungary	32 (0.4)	13 (0.3)
	Other	131 (1.7)	36 (0.9)
**Baseline EDSS - median (IQR)**^a^		2 (1, 2.5)	N/A
**Baseline EDSS - median (IQR)**^b^		2 (1, 2.5)	2 (1, 2.5)
**Baseline**^c^**MRI - T1 Gd** + **lesions - n (%)**	0	1263 (15.9)	971 (25.6)
	1+	825 (10.4)	658 (17.3)
	MRI performed, lesions not recorded	3117 (39.2)	2168 (57.1)
	No baseline MRI	2750 (34.6)	N/A
**Baseline**^c^**MRI - T2 lesions - n (%)**	0	22 (0.3)	14 (0.4)
	1–2	95 (1.2)	79 (2.1)
	3–8	884 (11.1)	649 (17.1)
	9+	1571 (19.8)	1220 (32.1)
	MRI performed, lesions not recorded	2633 (33.1)	1835 (48.3)
	No baseline MRI	2750 (34.6)	N/A
**First DMT - n (%)**	Rebif	2148 (27.0)	1046 (27.8)
	Betaferon	1754 (22.1)	690 (18.2)
	Avonex	1732 (21.8)	640 (16.9)
	Glatiramer acetate	1145 (14.4)	652 (17.2)
	Natalizumab	375 (4.7)	253 (6.7)
	Fingolimod	340 (4.3)	216 (5.7)
	DMF	186 (2.3)	127 (3.3)
	Teriflunomide	112 (1.4)	74 (2.0)
	Mitoxantrone	59 (0.7)	29 (0.8)
	Alemtuzumab	34 (0.4)	20 (0.5)
	Rituximab	22 (0.3)	12 (0.3)
	Cladribine	16 (0.2)	12 (0.3)
	Plegridy	15 (0.2)	12 (0.3)
	Daclizumab	12 (0.2)	10 (0.3)
	Ocrelizumab	5 (0.1)	4 (0.1)
**Relapses in the 12-months pre-baseline – mean (SD)**	–	–	1.21 (0.8)
**Relapses in the 12-months pre-baseline - median (IQR)**	–	–	1 (1, 2)

SD: standard deviation; IQR: interquartile range; EDSS: Expanded Disability Status Scale; DMT: disease-modifying treatment; MRI: magnetic resonance imaging.

a. Defined as both an EDSS and MRI recorded within 12 months of the diagnosis date.

a Defined as EDSS recorded closest to diagnosis within ±6 months (3775/7955).

b Defined as EDSS recorded closest to diagnosis within ±12 months (4304/7955).

c Defined as MRI recorded closest to diagnosis within ±12 months (5205/7955).

### Risk of clinical relapses

**Fig. 1:**
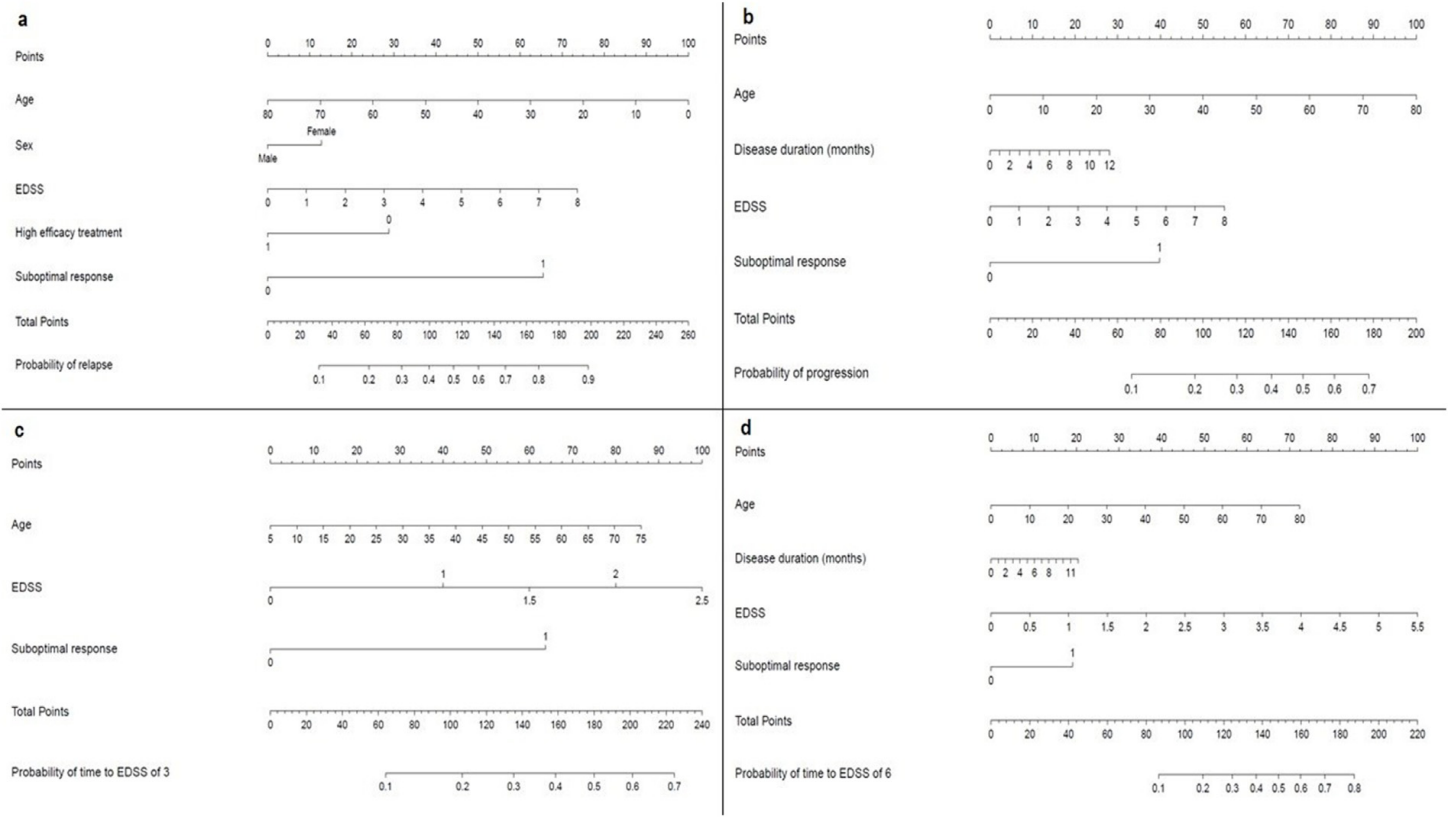
**Nomograms used to determine the risk of relapses, disability progression and achievement of EDSS milestones**. Each predictor has to be matched with the corresponding number of points on the top “Points” scale (vertical lines). a) Nomogram used to determine the risk of relapses within 5 years. b) Nomogram used to determine the risk of confirmed disability progression within 5 years. c) Nomogram used to determine the risk of reaching EDSS 3.0 within 5 years. d) Nomogram used to determine the risk of reaching EDSS 6.0 within 5 years. EDSS: Expanded Disability Status Scale.

Results from the multivariate analysis confirmed SR [HR 3.84 (95 ​% CI 3.51–4.19), p ​< ​0.001], female sex [HR 1.27 (95 ​% CI 1.16–1.40), p ​< ​0.001] and baseline EDSS [HR 1.19 (95 ​% CI 1.15–1.24), p ​< ​0.001] as independent risk factors for the occurrence of at least one clinical relapse within 5 years after the diagnosis of MS. HET as the first therapeutic choice [HR 0.78 (95 ​% CI 0.67–0.91), p ​= ​0.002] and an older age at baseline [HR 0.83 (95 ​% CI 0.79–0.86), p ​< ​0.001] were protective factors toward the explored outcome (Table 3; Table S2; Fig. 1). Results from subgroup analysis for patients on oral MET were reported in Table S11.

**Table 3:** Multivariate survival model for all outcomes.

Explanatory variable		First relapse	Disability progression	EDSS 3.0^c^	EDSS 6.0^d^	Conversion to SPMS	New brain MRI lesions	New spine MRI lesions	PIRA
	Category								
**Age at baseline (units** ​= ​**10 years)**		0.83 (0.79, 0.86) <**0.001**	0.92 (0.87, 0.97) **0.003**	1.20 (1.11, 1.30) <**0.001**	1.30 (1.17,1.45) <**0.001**	1.78 (1.51,2.10) <**0.001**	0.75 (0.70, 0.81) <**0.001**	0.83 (0.73, 0.94) **0.004**	1.89 (1.59, 2.24) <**0.001**
**Sex**	**Female**	1.27 (1.16, 1.40) <**0.001**	1.04 (0.93, 1.17) 0.471	1.03 (0.88, 1.22) 0.687	0.95 (0.76, .20) 0.681	0.61 (0.44, 0.85) **0.004**	1.02 (0.88, 1.17) 0.836	1.00 (0.78, 1.30) 0.974	–
	**Male**	Reference	Reference	Reference	Reference	Reference	Reference	Reference	–
**Months since first symptoms**		0.99 (0.98, 1.00) 0.071	0.98 (0.96, 0.99) **0.004**	1.02 (1.00, 1.05) **0.043**	1.03 (1.00,1.07) **0.041**	1.04 (0.99, 1.09) 0.107	0.99 (0.97, 1.01) 0.477	0.99 (0.95, 1.03) 0.534	1.07 (1.01, 1.12) **0.012**
**First DMT - high efficacy**	**Yes**	0.78 (0.67, 0.91) **0.002**	1.11 (0.93, 1.32) 0.243	0.99 (0.76, 1.30) 0.966	1.13 (0.80, .60) 0.484	0.84 (0.46, 1.53) 0.568	0.87 (0.70, 1.09) 0.240	0.95 (0.62, 1.44) 0.793	–
	**No**	Reference	Reference	Reference	Reference	Reference	Reference	Reference	–
**Baseline EDSS**		1.19 (1.15, 1.24) <**0.001**	1.18 (1.12, 1.23) <**0.001**	1.96 (1.75, 2.20) <**0.001**	1.61 (1.46,1.78) <**0.001**	1.31 (1.16, 1.48) <**0.001**	1.03 (0.96, 1.09) 0.427	1.00 (0.89, 1.12) 0.986	1.07 (0.94, 1.21) 0.304
**Pyramidal FS ​≥ ​2 - n (%)**	<**2**	Reference	Reference	Reference	Reference	Reference	Reference	Reference	–
	≥**2**	1.01 (0.89, 1.14) 0.925	0.97 (0.83, 1.13) 0.676	1.18 (0.93, 1.48) 0.168	1.39 (1.05,1.83) **0.023**	1.74 (1.13, 2.68) **0.012**	1.01 (0.83, 1.24) 0.885	1.25 (0.89, 1.74) 0.197	–
	**No baseline pyramidal KFS**	1.00 (0.88, 1.15) 0.958	0.72 (0.58, 0.88) **0.002**	1.01 (0.80, 1.28) 0.904	1.32 (0.80,0.60) 0.484	1.76 (1.10, 2.81) **0.018**	0.65 (0.50, 0.85) **0.002**	0.43 (0.23, 0.79) **0.007**	–
**Baseline brain MRI - T1 Gd ​+ lesions**	**0**	Reference	Reference	Reference	Reference	Reference	Reference	Reference	–
	**1**+	0.99 (0.87, 1.14) 0.927	0.99 (0.83, 1.17) 0.889	0.95 (0.75, 1.22) 0.696	1.13 (0.81,1.59) 0.470	1.18 (0.72, 1.93) 0.507	1.06 (0.86, 1.30) 0.598	0.83 (0.58, 1.119) 0.301	–
	**MRI performed, lesions not recorded**	0.97 (0.86, 1.09) 0.598	0.91 (0.78, 1.06) 0.234	1.07 (0.87, 1.32) 0.531	1.00 (0.74,1.34) 0.992	0.81 (0.52, 1.24) 0.331	1.04 (0.86, 1.26) 0.685	0.98 (0.69, 1.41) 0.930	–
**Baseline brain MRI - T2 lesions**	**0**	Reference	Reference	Reference	Reference	Reference	Reference	Reference	–
	**1–2**	1.73 (0.83, 3.62) 0.144	1.86 (0.62, 5.57) 0.270	0.75 (0.21, 2.69) 0.662	1.18 (0.28,4.89) 0.823	0.16 (0.01, 1.84) 0.142	2.44 (0.55, 10.75) 0.240	2.17 (0.25, 19.01) 0.483	–
	**3–8**	1.39 (0.70, 2.77) 0.344	1.69 (0.59, 4.87) 0.327	0.91 (0.28, 2.99) 0.875	1.10 (0.33,3.65) 0.882	0.38 (0.08, 1.75) 0.214	2.36 (0.56, 9.97) 0.242	1.06 (0.13, 8.73) 0.953	–
	**9**+	1.45 (0.73, 2.87) 0.289	1.67 (0.59, 4.79) 0.336	0.80 (0.25, 2.63) 0.719	0.92 (0.28,3.05) 0.889	0.53 (0.12, 2.38) 0.407	2.71 (0.64, 11.41) 0.174	1.13 (0.14, 9.18) 0.910	–
	**MRI performed, lesions not recorded**	1.52 (0.77, 3.02) 0.228	1.55 (0.54, 4.41) 0.417	0.87 (0.27, 2.84) 0.822	1.17 (0.36,3.83) 0.794	0.51 (0.12, 2.24) 0.371	1.91 (0.45, 8.06) 0.376	0.91 (0.11, 7.48) 0.931	–
**Sub-optimal response in first year of treatment**^a^	**Yes**^b^	3.84 (3.51, 4.19) <**0.001**	1.74 (1.56, 1.93) <**0.001**	3.01 (2.58, 3.51) <**0.001**	1.77 (1.43,2.20) <**0.001**	1.20 (0.87, 1.66) 0.258	2.33 (2.04, 2.66) <**0.001**	1.65 (1.29, 2.09) <**0.001**	–
	**No**	Reference	Reference	Reference	Reference	Reference	Reference	Reference	–

EDSS: Expanded Disability Status Scale; SPMS: secondary progressive multiple sclerosis; PIRA: progression independent from relapse activity; DMT: disease-modifying treatment; MRI: magnetic resonance imaging.

a Sub-optimal response ​= ​any new relapse OR new lesion OR EDSS increase during the first year of treatment.

b The relapse component considered as “sub-optimal response” were not considered as “first relapse” in this analysis.

c Subgroup analysis: it applies only to patients with a baseline EDSS<3.

d Subgroup analysis: it applies only to patients with a baseline EDSS<6.

### Confirmed disability worsening and improvement

**Fig. 2:**
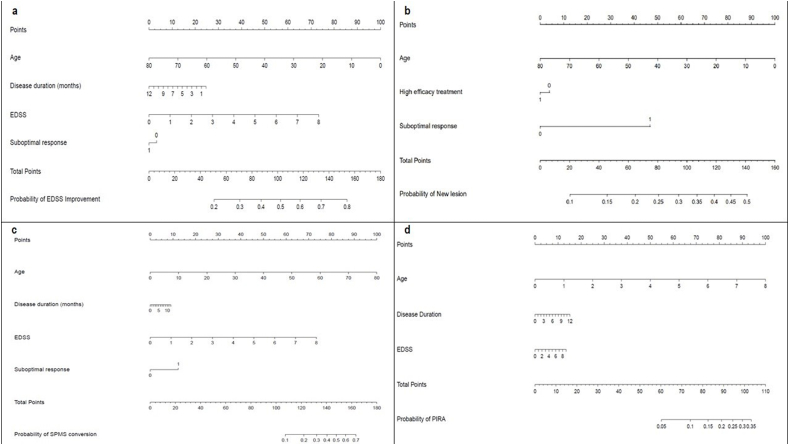
**Nomograms used to determine the risk of EDSS improvement, development of new brain lesions, conversion to SPMS and PIRA.** Each predictor has to be matched with the corresponding number of points on the top “Points” scale (vertical lines). a) Nomogram used to predict the risk of EDSS improvement within 5 years. b) Nomogram used to determine the risk of developing new brain lesions at MRI within 5 years. c) Nomogram used to determine the risk of conversion to SPMS within 5 years. d) Nomogram used to determine the risk of PIRA within 5 years. EDSS: Expanded Disability Status Scale; SPMS: Secondary Progressive Multiple Sclerosis; PIRA: Progression Independent of Relapse Activity.

In the overall study population, only a higher baseline EDSS [HR 1.18 (95 ​% CI 1.12–1.23), p ​< ​0.001] and SR [HR 1.74 (95 ​% CI 1.56–1.93), p ​< ​0.001] were associated with a significantly higher risk of disability worsening (Table 3; Table S3; Fig. 1). An older age at baseline [HR 0.78 (95 ​% CI 0.73–0.84), p ​< ​0.001] and longer disease duration [HR 0.95 (95 ​% CI 0.94–0.98), p ​< ​0.001], but not EDSS at baseline, were associated with a lower risk of confirmed disability improvement in the subgroup of patients with a baseline EDSS ≥2.0 (Table 3; Table S4; Fig. 2). Results from subgroup analyses for patients on oral MET were reported in Tables S12–S13.

### Reaching EDSS 3.0 and 6.0

SR [HR 3.01 (95 ​% CI 2.58–3.51), p ​< ​0.001], a higher EDSS at baseline [HR 1.96 (95 ​% CI 1.75–2.20), p ​< ​0.001], an older age at diagnosis [HR 1.20 (95 ​% CI 1.11–1.30), p ​< ​0.001] and a longer disease duration [HR 1.02 (95 ​% CI 1.00–1.05), p ​= ​0.043] were independent risk factors for the achievement of EDSS 3.0 within 5 years in patients who exhibited EDSS <3.0 ​at baseline (Table 3; Table S5; Fig. 1). The abovementioned variables were also significantly associated with the achievement of EDSS 6.0 (Table 3; Table S6; Fig. 1). The Pyramidal FS score ≥2 was a significant risk factor for EDSS milestone 6.0 [HR 1.39 (95 ​% CI 1.05–1.83), p ​= ​0.023], but not for EDSS milestone 3.0. The number of T2 and Gd ​+ ​lesions at brain and spinal MRI did not predict the achievement of EDSS 3.0 or 6.0. Subgroup analyses for patients on oral MET were not conducted for these outcomes due to insufficient sample.

### Conversion to SPMS

The main predictors for the risk of conversion into SPMS were age [HR 1.78 (95 ​% CI 1.51–2.10), p ​< ​0.001] and EDSS [HR 1.31 (95 ​% CI 1.16–1.48), p ​< ​0.001] at baseline, and Pyramidal FS score ≥2 [HR 1.74 (95 ​% CI 1.13–2.68), p ​= ​0.012]. Conversely, the female sex was a protective factor for the explored outcome [HR 0.61 (95 ​% CI 0.44–0.85), p ​= ​0.004] (Table 3; Table S7; Fig. 2). Subgroup analysis for patients on oral MET was not conducted for this outcome due to insufficient sample.

### Development of new brain or spinal lesions at MRI scans

The risk of detecting new lesions at brain MRI scans was lower in patients who were older at the time of diagnosis [HR 0.75 (95 ​% CI 0.70–0.81), p ​< ​0.001] and higher in patients exhibiting SR [HR ​= ​2.33 (95 ​% CI 2.04–2.66), p ​< ​0.001] (Table 3; Table S8). Similarly, the aforementioned variables predicted the occurrence of new lesions at spinal MRI (Table 3; Table S9; Fig. 2). Results from subgroup analysis for patients on oral MET were reported in Tables S14–15.

### PIRA

Among all variables explored, only age [HR 1.89 (95 ​% CI 1.59–2.24), p ​< ​0.001] and EDSS at baseline [HR 1.07 (95 ​% CI 1.02–1.12), p ​= ​0.012] were independent risk factors for the development of PIRA (Table 3; Table S10; Fig. 2). The number of T2 and Gd ​+ ​lesions at brain and spinal MRI were not predictive for the explored outcome. Results from subgroup analysis for patients on oral MET were reported in Table S16.

## Discussion

Our study confirms the crucial role of the first therapeutic choice and early treatment response on the 5-year prognosis of patients with MS.

It is known that the immediate initiation of HET is preferable to treatment escalation strategy in reducing the rate of relapses and disability progression [15]. Further, the timing for the introduction of HET seems to be equally important. Data from the MSBase registry and Swedish MS registry confirmed that HET started within 2 years from disease onset is protective toward the development of disability within 6–10 years [16]. Additionally, an Italian MS Registry study assessed the effects of early and late start of HET in patients with RRMS, reporting significantly higher mean annual delta-EDSS values in the escalation group compared with the early intensive treatment group at all timepoints and more markedly in the long-term, up to 10 years [17].

Our results confirmed that early treatment response to the first therapeutic choice is a predictor for almost all outcomes explored. In this regard, a sub-optimal response within the first year of treatment was associated with an increased risk more than 3-fold for relapses and 2-fold for developing new brain lesions at MRI scans. Additionally, an incomplete response to the first DMT not only predicted clinical and radiological signs of disease activity, but was also associated with a higher risk of disease progression (HR ​= ​1.74) and achievement of EDSS 3.0 (HR ​= ​3.01) and 6.0 (HR ​= ​1.77). This is particularly relevant, considering the two-stage model for disability progression proposed by Leray and colleagues [11]. In this view, demographic and clinical factors can only affect the time needed to reach EDSS 3.0, while the disability progression from this milestone to EDSS 6.0 lasted from 6 to 9 years irrespective of the previous phase duration. As a consequence, efforts should be concentrated in delaying the achievement of EDSS 3.0. In our study, a sub-optimal treatment response in the first year after treatment start was the most relevant independent predictor for reaching EDSS 3.0, being associated with a 3-fold higher risk to achieve the outcome within 5 years from the time of diagnosis.

An older age at the time of diagnosis and a higher EDSS at baseline were also predictive for conversion to SPMS, EDSS milestones 3.0 and 6.0, in line with results from previous studies [18]. On the other hand, an older age at baseline was a protective factor toward clinical and radiological activity, reducing by 25 ​% the risk of relapses and detection of new brain MRI lesions within 5 years. Recent data reported a decrease in clinical and subclinical disease activity, as shown in our study, together with a lower efficacy of DMT and poor post-relapse recovery with aging, most likely due to immune-senescence [19,20]. An older age at baseline was associated with an increased risk of converting to SPMS within 5 years in our study, confirming evidence of common onset of the progressive phase in MS in the fifth decade [19]. Our results confirmed the role of sex in affecting disease activity and progression [21]. Indeed, female sex was a risk factor for the occurrence of relapses within the first 5 years from diagnosis, confirming the higher frequency of autoimmune responses in women. However, female sex was a protective factor toward the transition into SPMS. Previous studies reported shorter times to achieve given disability levels and to convert into SPMS from MS onset in men compared with women [[22], [23], [24], [25]].

In our study, the risk of conversion to SPMS within 5 years was also predicted by an older age and a higher EDSS at baseline, as well as by male sex, but not by sub-optimal response in the first year of treatment. Despite several studies exploring predictive factors of conversion to SPMS have not been conclusive as yet, most reported results similar to ours [[26], [27], [28], [29]]. Particularly, older age seems to increase the risk of progression to SPMS regardless of disease duration [27]. It should be noted that universally accepted criteria for SPMS diagnosis do not yet exist and that, in our study, different criteria were probably used by MS Centers to establish the timing of SPMS diagnosis. However, the aforementioned predictors were corroborated in our analysis when adjusting the model for inter-clinic heterogeneity, except for SR, whose association with an increased risk for transition to SPMS was not confirmed.

The difficulty in identifying the moment of transition from RRMS to SPMS remains a major challenge and can cause a diagnostic delay of up to 3 years, due to our inadequate measuring tools (e.g. EDSS) [27]. Indeed, the traditional biphasic view of MS, as mainly characterized by inflammation before and neurodegeneration later, is being questioned by new evidence and modern imaging techniques. Imaging markers of chronic inflammation, as slowly expanding lesions, paramagnetic rim sign, and microglial activation, are already present in the relapsing-remitting phase [[30], [31], [32]], as well as the histopathological evidence of axonal damage [33], documenting a “silent progression” occurring in patients who meet the criteria for RRMS [34]. PIRA seems to be the main driver of disease worsening in all phenotypes of MS, and can start even after the first demyelinating event [2,4].

Thus, it seems that MS progresses as a continuum from relapsing to progressive disease and progression, even if difficult to identify, is present from the very early phases of the disease. We investigated the impact of the variables explored on PIRA. Age and disease duration from symptoms onset were the only predictors in our model, with a greater impact exerted by age [HR ​= ​1.89]. This highlights the need to better understand the underlying mechanisms and to develop tools and new biomarkers which can be sensitive in detecting insidious disease progression. Of note, HET did not reduce the risk of PIRA, as well as SR, suggesting that a change in the therapeutic approach could be needed, including the potential use of combination therapies.

**Fig. 3:**
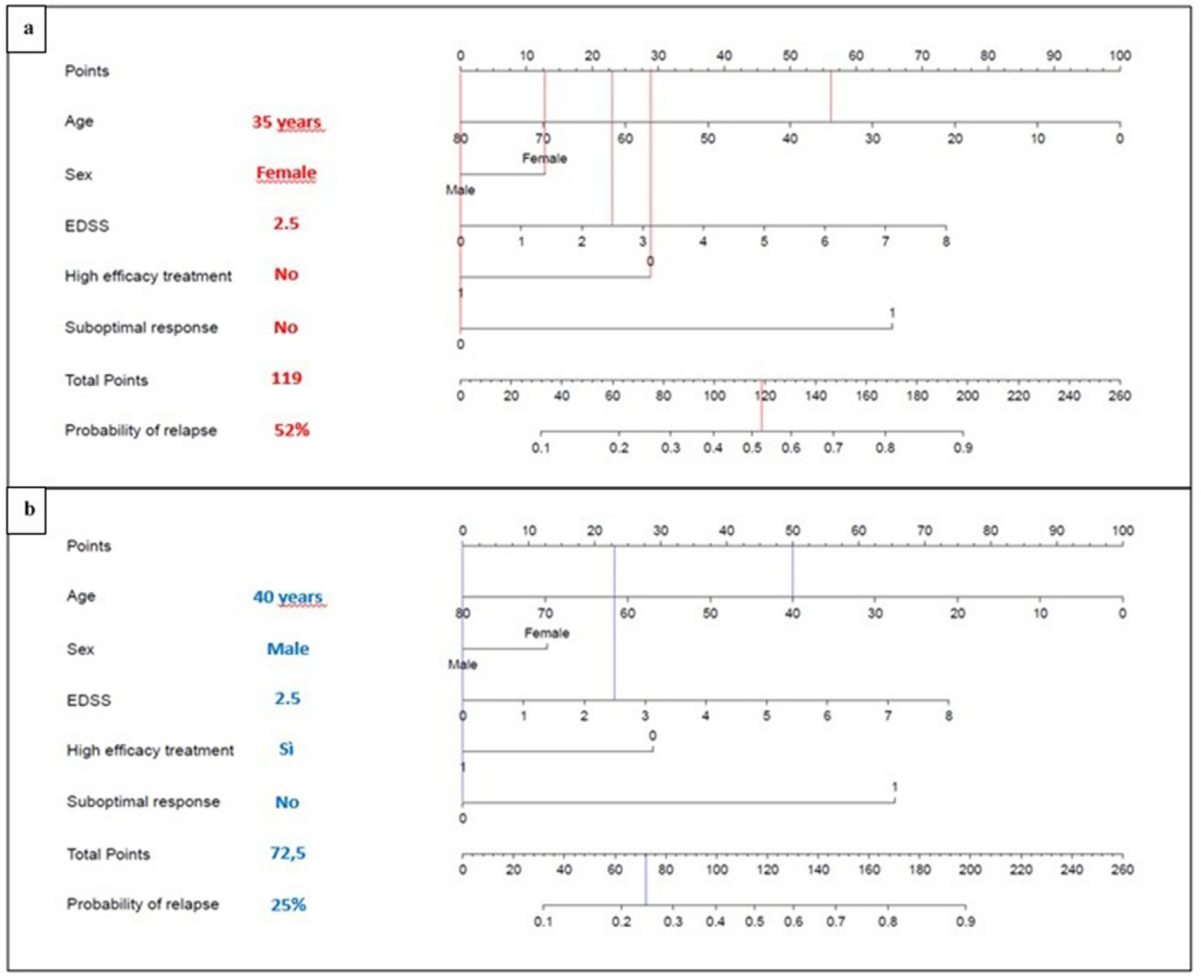
**Worked example of how to use nomograms to predict the risk of relapses during the first 5 years from diagnosis.** Each predictor has to be matched with the corresponding number of points on the top “Points” scale (vertical lines). a) The age of 35 years matches to 56 points, the female sex to 12.5 points, a baseline EDSS score of 2.5 matches to 22.5 points, the choice of DMT others than HET corresponds to 28 points and the absence of suboptimal response to 0 points. This sums to a cumulative total of 119 points. Drawing a line down from the “Total Points” scale to the corresponding “Probability of relapse” scale reveals that 119 total points corresponds to a probability of relapses of 52 ​% for this hypothetic patient. b) The age of 40 years (50 points), the male sex (0 points), a baseline EDSS score of 2.5 (22.5 points), the use of HET (0 points) and the absence of suboptimal response (0 points) sums to a cumulative score of 72.5 points, corresponding to a 25 ​% probability of relapses for this hypothetic patient.

For each outcome, nomograms were built including the significant predictors among all variables explored (Fig. 1, Fig. 2). Nomograms can provide a useful support in the decision-making process of MS management, allowing clinicians to obtain rapid and personalized risk estimates and thus facilitating patient therapeutic counseling. The risk estimates can be easily obtained by drawing vertical lines from each predictor upwards to the point axis, adding up the partial scores and drawing a vertical line from the total point axis downwards to the outcome probability axis. We hypothesized two different clinical scenarios to better explain the use of nomograms. For example, a hypothetic 35-year-old female patient, with baseline EDSS score of 2.5 and early optimal response to MET, would exhibit a 52 ​% risk of relapses during the first 5 years, as illustrated in Fig. 3. Differently, a supposed 40-year- old male subject, with comparable EDSS and optimal response to HET, would experience a risk of 25 ​% of clinical relapses within 5 years.

Even if the number of predictors included is limited, this can represent an advantage in terms of facilitated use in clinical practice, since all the variables considered are easily accessible during a routine neurological visit. In a recent study, machine learning algorithms were used to identify predictors for several disability outcomes, including the risk of confirmed disability worsening [35]. Despite proteomics was also used as a potential predictor, results from this study supported the use of clinical and imaging data commonly collected at the outpatient clinic, since these ones allowed to achieve a good accuracy with easily available information. Even if few previous studies reported nomograms as a valid tool to predict the risk of specific outcomes [36,37], and others proposed models to early predict conversion to SPMS [38,39], to our knowledge this is the first one which combines multiple and commonly achievable potential prognostic factors focusing on the impact of the first therapeutic choice and of the very early treatment response on a mid-long term prognosis for patients with MS.

This study exhibits some limitations. First, the incomplete reporting of data in iMed©, particularly MRI data and FS scores, almost certainly affected the results of the analysis. Indeed, the number of T2 and Gd ​+ ​brain and spine lesions often were not recorded and were not retrievable to be used in the analysis. Although the number of MRI scans per patient in the analyzed sample was reasonable (averaging 1.30 scans per patient per year of follow-up), MRI data were not part of the MSBase minimum dataset, unlike clinical data as EDSS score. Thus, complete and partial missing lesion data remains a significant limitation of this study. Additionally, lesion volumes and measures of brain atrophy were not available. This could explain why MRI data and the Pyramidal FS score, which have been reported as relevant prognostic factors in previous studies [28,40], did not reach statistical significance in our model and were not included in nomograms. Second, despite data are inserted real-time in iMed©, the study is observational and extends over a long period of time, when diagnostic criteria have been revised more than once, progressively increasing in sensitivity. Another relevant aspect includes the predominant use of oral MET as first therapeutic option compared to injectables. For this reason, we conducted subgroup analyses limited to patients on oral MET for all outcomes, despite the small sample size limited the generalizability of the results.

Finally, the requirement for a minimum 5-years follow-up could introduce selection bias toward those patients with greater disability who stopped attending clinic and the ones who continued the clinical follow-up.

Our study provides further evidence about the crucial role played by the initial treatment response to the first therapeutic approach, independently of the considered country, epoch and clinic. In addition, it confirms the relevance of demographic and clinical factors on the mid-term prognosis of patients with MS. This can be considered as a first step, in the expectation of conducting external validation of results in separate cohort.

A highly effective approach since the time of diagnosis is warranted, especially in patients with adverse prognostic factors, and risk stratification of patients with MS in every day practice may be guided by simple prognostic tools, as nomograms, procedural flowcharts and risk tables.

## Informed consent

All subjects involved in the study provided informed consent to allow data collection and the use of clinical data for study purpose.

## Ethical standards

The study was conducted according to the guidelines of the Declaration of Helsinki of 1964 and later amendments, and approved by the Ethics Committee of the A.O.U. Policlinico-San Marco of Catania and the Melbourne Health Human Research Ethics Committee.

## Author contributions

*Concept and design:* Simona Toscano, Francesco Patti. *Acquisition, analysis, or interpretation of data:* Simona Toscano, Francesco Patti, Tim Spelman. *Drafting of the manuscript:* Simona Toscano, Francesco Patti, Tim Spelman. *Critical revision of the manuscript for important intellectual content:* Simona Toscano, Francesco Patti, Tim Spelman, Serkan Ozakbas, Raed Alroughani, Clara G. Chisari, Salvatore Lo Fermo, Alexandre Prat, Marc Girard, Pierre Duquette, Guillermo Izquierdo, Sara Eichau, Pierre Grammond, Cavit Boz, Tomas Kalincik, Yolanda Blanco, Katherine Buzzard, Olga Skibina, Maria Jose Sa, Anneke van der Walt, Helmut Butzkueven, Murat Terzi, Oliver Gerlach, Francois Grand'Maison, Matteo Foschi, Andrea Surcinelli, Michael Barnett, Alessandra Lugaresi, Marco Onofrj, Bassem Yamout, Samia J. Khoury, Julie Prevost, Jeannette Lechner-Scott, Davide Maimone, Maria Pia Amato, Daniele Spitaleri, Vincent Van Pesch, Richard Macdonell, Elisabetta Cartechini, Koen de Gans, Mark Slee, Tamara Castillo-Triviño, Aysun Soysal, Yara Fragoso†, Jose Luis Sanchez-Menoyo, Guy Laureys, Liesbeth Van Hijfte, Pamela McCombe, Ayse Altintas, Bianca Weinstock-Guttman, Eduardo Aguera-Morales, Masoud Etemadifar, Cristina Ramo-Tello, Gerardo Iuliano, Nevin John, Recai Turkoglu, Suzanne Hodgkinson, Sarah Besora, Bart Van Wijmeersch, Ricardo Fernandez Bolaños. *Statistical analysis:* Tim Spelman. *Administrative, technical, or material* support*:* Tim Spelman, Tomas Kalincik. *Supervision:* Simona Toscano, Francesco Patti, Tim Spelman.

## Funding

No funding was received toward this work.

## Declaration of competing interest

The authors declare the following financial interests/personal relationships which may be considered as potential competing interests: Dr Spelman, Dr Ozakbas, Dr Prat, Dr Girard, Dr Gerlach, Dr Surcinelli, Dr Onofrj, Dr Cartechini, Dr Slee, Dr Soysal, Dr Etemadifar, Dr Turkoglu and Dr Besora have nothing to declare. Dr Toscano has received congress and travel/accommodation from Biogen, Novartis, Roche, Janssen and Sanofi Genzyme. Dr Alroughani received honoraria as a speaker and for serving on scientific advisory boards from Bayer, Biogen, GSK, Merck, Novartis, Roche and Sanofi-Genzyme. Dr Chisari has received congress and travel/accommodation from Alexion, Almirall, Biogen, Merck Serono, Novartis, Roche, and Sanofi Genzyme. Dr Lo Fermo has received congress and travel/accommodation from Alexion, Almirall, Biogen, Merck Serono, Novartis, Roche, and Sanofi Genzyme. Dr Duquette served on editorial boards and has been supported to attend meetings by EMD, Biogen, Novartis, Genzyme, and TEVA Neuroscience; he holds grants from the CIHR and the MS Society of Canada and has received funding for investigator-initiated trials from Biogen, Novartis, and Genzyme. Dr Izquierdo received speaking honoraria from Biogen, Novartis, Sanofi, Merck, Roche, Almirall and Teva. Dr Eichau received speaker honoraria and consultant fees from Biogen Idec, Novartis, Merck, Bayer, Sanofi Genzyme, Roche and Teva. Dr Grammond has served in advisory boards for Novartis, EMD Serono, Roche, Biogen idec, Sanofi Genzyme, Pendopharm and has received grant support from Genzyme and Roche, has received research grants for his institution from Biogen idec, Sanofi Genzyme, EMD Serono. Dr Boz received conference travel support from Biogen, Novartis, Bayer-Schering, Merck and Teva; has participated in clinical trials by Sanofi Aventis, Roche and Novartis. Dr Kalincik served on scientific advisory boards for MS International Federation and World Health Organisation, BMS, Roche, Janssen, Sanofi Genzyme, Novartis, Merck and Biogen, steering committee for Brain Atrophy Initiative by Sanofi Genzyme, received conference travel support and/or speaker honoraria from WebMD Global, Eisai, Novartis, Biogen, Roche, Sanofi-Genzyme, Teva, BioCSL and Merck and received research or educational event support from Biogen, Novartis, Genzyme, Roche, Celgene and Merck. Dr Blanco received speaker honoraria/consulting fees from Merck, Biogen, Roche, Brystol, Novartis, Sanofi and Sandoz. Dr Buzzard received speaker honoraria and/or education support from Biogen, Teva, Novartis, Genzyme-Sanofi, Roche, Merck and Alexion; has been a member of advisory boards for Merck and Biogen. Dr Skibina received honoraria and consulting fees from Bayer Schering, Novartis, Merck, Biogen and Genzyme. Dr Sa received consulting fees, speaker honoraria, and/or travel expenses for scientific meetings from Alexion, Bayer Healthcare, Biogen, Bristol Myers Squibb, Celgene, Janssen, Merck-Serono, Novartis, Roche, Sanofi and Teva. Dr van der Walt served on advisory boards and receives unrestricted research grants from Novartis, Biogen, Merck and Roche She has received speaker's honoraria and travel support from Novartis, Roche, and Merck; she receives grant support from the National Health and Medical Research Council of Australia and MS Research Australia. Dr Butzkueven received institutional (Monash University) funding from Biogen, F. Hoffmann-La Roche Ltd, Merck, Alexion, CSL, and Novartis; has carried out contracted research for Novartis, Merck, F. Hoffmann-La Roche Ltd and Biogen; has taken part in speakers' bureaus for Biogen, Genzyme, UCB, Novartis, F. Hoffmann-La Roche Ltd and Merck; has received personal compensation from Oxford Health Policy Forum for the Brain Health Steering Committee. Dr Terzi received travel grants from Novartis, Bayer-Schering, Merck and Teva; has participated in clinical trials by Sanofi Aventis, Roche and Novartis. Dr Grand’Maison received honoraria or research funding from Biogen, Genzyme, Novartis, Teva Neurosciences, and ATARA Pharmaceuticals. Dr Foschi received travel and meeting attendance support from Novartis, Biogen, Roche, Sanofi-Genzyme and Merck. Dr Barnett served on scientific advisory boards for Biogen, Novartis and Genzyme and has received conference travel support from Biogen and Novartis; he serves on steering committees for trials conducted by Novartis. His institution has received research support from Biogen, Merck and Novartis. Dr Lugaresi has received personal compensation for consulting, serving on a scientific advisory board, speaking or other activities from Alexion, Biogen, Bristol Myers Squibb, Janssen, Merck Serono, Novartis, and Sanofi/Genzyme, and Her institutions have received research grants from Novartis and Sanofi/Genzyme. Dr Yamout received honoraria as a speaker and member of scientific advisory boards from Sanofi, Bayer, Biogen, Merck, Janssen, Novartis, Roche and Aspen. Dr Khoury received compensation for scientific advisory board activity from Merck and Roche, and received compensation for serving on the IDMC for Biogen. Dr Prevost accepted travel compensation from Novartis, Biogen, Genzyme, Teva, and speaking honoraria from Biogen, Novartis, Genzyme and Teva. Dr Lechner-Scott received travel compensation from Novartis, Biogen, Roche and Merck. Her institution receives the honoraria for talks and advisory board commitment as well as research grants from Biogen, Merck, Roche, TEVA and Novartis. Dr Maimone received speaker honoraria for Advisory Board and travel grants from Almirall, Biogen, Merck, Novartis, Roche, Sanofi-Genzyme, and Teva. Dr Amato received honoraria as consultant on scientific advisory boards by Biogen, Bayer-Schering, Merck, Teva and Sanofi-Aventis; has received research grants by Biogen, Bayer-Schering, Merck, Teva and Novartis. Dr Spitaleri received honoraria as a consultant on scientific advisory boards by Bayer-Schering, Novartis and Sanofi-Aventis and compensation for travel from Novartis, Biogen, Sanofi Aventis, Teva and Merck. Dr van Pesch received travel grants from Merck Healthcare KGaA (Darmstadt, Germany), Biogen, Sanofi, Bristol Meyer Squibb, Almirall and Roche. His institution has received research grants and consultancy fees from Roche, Biogen, Sanofi, Merck Healthcare KGaA (Darmstadt, Germany), Bristol Meyer Squibb, Janssen, Almirall, Novartis Pharma, and Alexion. Dr Macdonell or his institution have received remuneration for his speaking engagements, advisory board memberships, research and travel from Biogen, Merck, Genzyme, Bayer, Roche, Teva, Novartis, CSL, BMS, MedDay and NHMRC. Dr de Gans served on scientific advisory boards for Roche, Janssen, Sanofi-Genzyme, Novartis and Merck, received conference fee and travel support from Novartis, Biogen, Sanofi-Genzyme, Teva, Abbvie and Merck and received educational event support from Novartis. Dr Castillo-Triviño received speaking/consulting fees and/or travel funding from Almirall, Biogen, Bristol Myers Squibb, Janssen, Merck, Novartis, Roche, Sanofi-Genzyme and Teva. Dr Sanchez-Menoyo accepted travel compensation from Novartis, Merck and Biogen, speaking honoraria from Biogen, Novartis, Sanofi, Merck, Almirall, Bayer and Teva and has participated in clinical trials by Biogen, Merck and Roche. Dr laureys received travel and/or consultancy compensation from Sanofi-Genzyme, Roche, Teva, Merck, Novartis, Celgene, Biogen. Dr Van Hijfte received travel compensation from Merck. Dr McCombe received speaker fees and travel grants from Novartis, Biogen, T’évalua, Sanofi. Dr Altintas received speaker honoraria from Novartis and Alexion. Dr Weinstock-Guttman has participated in speaker's bureaus and/or served as a consultant for Biogen, EMD Serono, Novartis, Genentech, Celgene/Bristol Meyers Squibb, Sanofi Genzyme, Bayer, Janssen and Horizon; he has received grant/research support from the agencies listed in the previous sentence. She serves in the editorial board for BMJ Neurology, Children, CNS Drugs, MS International and Frontiers Epidemiology. Dr Aguera-Morales has received honoraria as a speaker and for serving on scientific advisory boards from Biogen, Merck, Novartis and Sanofi-Genzyme. Dr Ramo-Tello has received research funding, or compensation for consulting services and speaker honoraria, or meetings travels from Biogen, Novartis, Sanofi, Bristol, Roche, Almirall and Merck. Dr John received funding from Novartis, Biogen, Amicus and Sanofi. Dr Hodgkinson has received consulting fees and speaker honoraria from Biogen, Novartis, Roche, Merck, and has received grants for her Institution from Biogen, Merck, Novartis, and Roche. Dr Van Wijmeersch received research and travel grants, honoraria for MS-Expert advisor and Speaker fees from Almirall, Biogen, BMS, Imcyse, Janssen, Sanofi, Merck, Novartis, Roche and Teva. Dr Fernandez-Bolaños received speaking honoraria from Biogen, Novartis, Merck and Teva. Prof. Patti has received honoraria for speaking activities by Alexion, Almirall, Bayer Schering, Biogen, Merck Serono, Novartis, Roche, Sanofi Genzyme, and TEVA; he also served as advisory board member for the following companies: Bayer Schering, Biogen, Merck Serono, Novartis, Roche, Sanofi Genzyme, and TEVA; he was funded by Pfizer and FISM for epidemiological studies; he received grants for congress participation from Alexion, Almirall, Bayer Shering, Biogen, Merck Serono, Novartis, Roche, Sanofi Genzyme, and TEVA. If there are other authors, they declare that they have no known competing financial interests or personal relationships that could have appeared to influence the work reported in this paper.
