# Supplementary material for: First-year treatment response predicts the following 5-year disease course in patients with relapsing-remitting multiple sclerosis
Source: Neurotherapeutics. 2025 Feb 17;22(2):e00552. doi: 10.1016/j.neurot.2025.e00552 (PMC12014414; doi:10.1016/j.neurot.2025.e00552)
Supplement: Multimedia component 1 [file mmc1.docx]

**Table S1.** Definitions for the outcomes explored in the study population.

| **Primary outcomes** | **Definitions** |
| --- | --- |
| **Clinical relapses** | Occurrence of at least one episode of new neurological symptoms, or the return of old symptoms, for a period of 24 hours or more, in the absence of an infection or a change in core body temperature. |
| **Disability worsening** | Increase in EDSS score confirmed at 24 weeks, using the baseline EDSS score as reference^1^ |
| **Conversion to SPMS** | Diagnosis of SPMS is usually defined based on the deterioration independent of relapses for ≥6 months following an initial relapsing-remitting course. Different criteria could have been used by the recruiting MS Centers. |
| **PIRA** | Confirmed disability accumulation in the EDSS scale at 6 months, as compared with baseline EDSS, during a period free of relapses. A period free of relapses is defined as the time between 2 consecutive relapses, starting 3 months after a relapse. |
| **Secondary outcomes** |  |
| **Disability Improvement** | Decrease in EDSS score confirmed at 24 weeks, using the baseline EDSS score as reference. Only applies to patients with a baseline EDSS ≥ 2.0. |
| **EDSS 3.0** | Reaching a sustained EDSS milestone 3.0 (moderate disability but no impairment of walking). Only applies to patients with baseline EDSS < 3.0 |
| **EDSS 6.0** | Reaching a sustained EDSS milestone 6.0 (unilateral assistance required to walk about 100 m with or without resting). Only applies to patients with baseline EDSS < 6.0 |
| **New brain MRI lesions** | Detection of new focal areas of high signal intensity in T2WI/T2-FLAIR sequences at brain MRI, compared with the previous MRI scan. |
| **New spine MRI lesions** | Detection of new focal areas of high signal intensity in T2WI/T2-FLAIR sequences at spinal cord MRI, compared with the previous MRI scan. |
