# Supplementary material for: First-year treatment response predicts the following 5-year disease course in patients with relapsing-remitting multiple sclerosis
Source: Neurotherapeutics. 2025 Feb 17;22(2):e00552. doi: 10.1016/j.neurot.2025.e00552 (PMC12014414; doi:10.1016/j.neurot.2025.e00552)
Supplement: Multimedia component 2 [file mmc2.docx]

**Table S2.** Risk of relapses within 5 years from diagnosis

|  |  | **Univariate, Random effects = country & epoch** | **Multivariate, Random effects = country & epoch** | **Multivariate, Random effects = country, epoch & clinic** |
| --- | --- | --- | --- | --- |
| **Explanatory variable** | **Category** | **Hazard Ratio (95% CI) p-value** | **Hazard Ratio (95% CI) p-value** | **Hazard Ratio (95% CI) p-value** |
| Age at baseline (units=10 years) |  | **0.85 (0.81, 0.89)**  **<0.001** | **0.83 (0.79, 0.86) <0.001** | **0.83 (0.79, 0.86) <0.001** |
| Sex | Female | **1.19 (1.08, 1.30)**  **<0.001** | **1.27 (1.16, 1.40) <0.001** | **1.27 (1.16, 1.40) <0.001** |
|  | Male | Reference | Reference | Reference |
|  | Not recorded | Insufficient events | Insufficient events | Insufficient events |
| Months since first symptoms |  | **0.99 (0.97, 1.00)**  **0.036** | **0.99 (0.97, 1.00) 0.045** | 0.99 (0.98, 1.00) 0.071 |
| First DMT - high efficacy | Yes | 0.88 (0.76, 1.03)  0.106 | **0.79 (0.67, 0.92) 0.002** | **0.78 (0.67, 0.91) 0.002** |
|  | No | Reference | Reference | Reference |
| Baseline EDSS |  | **1.10 (1.07, 1.13)**  **<0.001** | **1.19 (1.15, 1.23) <0.001** | **1.19 (1.15, 1.24) <0.001** |
| Baseline Pyramidal KFS ≥ 2 - n (%) | <2 | Reference | Reference | Reference |
|  | ≥2 | **1.23 (1.11, 1.36)**  **<0.001** | 1.02 (0.90, 1.15) 0.811 | 1.01 (0.89, 1.14) 0.925 |
|  | No baseline pyramidal KFS | 1.02 (0.91, 1.15) 0.716 | 1.01 (0.89, 1.14) 0.886 | 1.00 (0.88, 1.15) 0.958 |
| Baseline Brain MRI - T1 Gd+ lesions | 0 | Reference | Reference | Reference |
|  | 1+ | 1.07 (0.94, 1.22)  0.280 | 1.04 (0.92, 1.19) 0.512 | 0.99 (0.87, 1.14) 0.927 |
|  | MRI performed, lesions not recorded | 0.98 (0.88, 1.09)  0.694 | 0.96 (0.86, 1.08) 0.495 | 0.97 (0.86, 1.09) 0.598 |
| Baseline Brain MRI - T2 lesions | 0 | Reference | Reference | Reference |
|  | 1-2 | 1.00 (0.49, 2.05)  0.995 | 1.58 (0.77, 3.24) 0.215 | 1.73 (0.83, 3.62) 0.144 |
|  | 3-8 | 0.93 (0.48, 1.82)  0.833 | 1.32 (0.68, 2.59) 0.412 | 1.39 (0.70, 2.77) 0.344 |
|  | 9+ | 0.92 (0.47, 1.80)  0.815 | 1.30 (0.67, 2.54) 0.440 | 1.45 (0.73, 2.87) 0.289 |
|  | MRI performed, lesions not recorded | 0.92 (0.47, 1.79)  0.803 | 1.45 (0.75, 2.83) 0.273 | 1.52 (0.77, 3.02) 0.228 |
| Sub-optimal response in first year of treatment* | Yes | **3.55 (3.26, 3.86)**  **<0.001** | **3.77 (3.46, 4.11) <0.001** | **3.84 (3.51, 4.19) <0.001** |
|  | No | Reference | Reference | Reference |

* sub-optimal response = any new relapse OR new lesion OR EDSS increase during the first year of treatment
