# Supplementary material for: First-year treatment response predicts the following 5-year disease course in patients with relapsing-remitting multiple sclerosis
Source: Neurotherapeutics. 2025 Feb 17;22(2):e00552. doi: 10.1016/j.neurot.2025.e00552 (PMC12014414; doi:10.1016/j.neurot.2025.e00552)
Supplement: Multimedia component 3 [file mmc3.docx]

**Table S3.** Risk of disability worsening within 5 years from diagnosis

|  |  | **Univariate, Random effects = country & epoch** | **Multivariate, Random effects = country & epoch** | **Multivariate, Random effects = country, epoch & clinic** |
| --- | --- | --- | --- | --- |
| **Explanatory variable** | **Category** | **Hazard Ratio (95% CI) p-value** | **Hazard Ratio (95% CI) p-value** | **Hazard Ratio (95% CI) p-value** |
| Age at baseline (units=10 years) |  | **1.26 (1.16, 1.36) <0.001** | **1.19 (1.10, 1.29) <0.001** | **0.92 (0.87, 0.97) 0.003** |
| Sex | Female | 1.05 (0.88, 1.26) 0.575 | 1.06 (0.88, 1.26) 0.559 | 1.04 (0.93, 1.17) 0.471 |
|  | Male | Reference | Reference | Reference |
|  | Not recorded | Insufficient events | Insufficient events | Insufficient events |
| Months since first symptoms |  | **1.04 (1.02, 1.06) 0.001** | **1.03 (1.01, 1.06) 0.012** | **0.98 (0.96, 0.99) 0.004** |
| First DMT - high efficacy | Yes | 1.17 (0.91, 1.50) 0.219 | 1.12 (0.89, 1.42) 0.336 | 1.11 (0.93, 1.32) 0.243 |
|  | No | Reference | Reference | Reference |
| Baseline EDSS |  | **1.31 (1.24, 1.39) <0.001** | **1.25 (1.16, 1.34) <0.001** | **1.18 (1.12, 1.23) <0.001** |
| Baseline Pyramidal KFS ≥ 2 - n (%) | <2 | Reference | Reference | Reference |
|  | ≥2 | **2.01 (1.66, 2.43) <0.001** | 1.23 (0.98, 1.56) 0.078 | 0.97 (0.83, 1.13) 0.676 |
|  | No baseline pyramidal KFS | 1.17 (0.94, 1.46) 0.161 | 1.08 (0.86, 1.36) 0.492 | **0.72 (0.58, 0.88) 0.002** |
| Baseline Brain MRI - T1 Gd+ lesions | 0 | Reference | Reference | Reference |
|  | 1+ | 0.93 (0.73, 1.20) 0.598 | 0.95 (0.74, 1.23) 0.710 | 0.99 (0.83, 1.17) 0.889 |
|  | MRI performed, lesions not recorded | 0.85 (0.70, 1.03) 0.100 | 0.83 (0.68, 1.03) 0.085 | 0.91 (0.78, 1.06) 0.234 |
| Baseline Brain MRI - T2 lesions | 0 | Reference | Reference | Reference |
|  | 1-2 | 1.27 (0.37, 4.38) 0.704 | 1.81 (0.53, 6.23) 0.347 | 1.86 (0.62, 5.57) 0.270 |
|  | 3-8 | 0.98 (0.31, 3.11) 0.977 | 1.37 (0.43, 4.32) 0.596 | 1.69 (0.59, 4.87) 0.327 |
|  | 9+ | 0.86 (0.27, 2.70) 0.796 | 1.20 (0.38, 3.79) 0.750 | 1.67 (0.59, 4.79) 0.336 |
|  | MRI performed, lesions not recorded | 0.89 (0.28, 2.78) 0.840 | 1.38 (0.44, 4.32) 0.579 | 1.55 (0.54, 4.41) 0.417 |
| Sub-optimal response* in first year of treatment | Yes | **2.49 (2.11, 2.94) <0.001** | **2.56 (2.16, 3.02) <0.001** | **1.74 (1.56, 1.93) <0.001** |
|  | No | Reference | Reference | Reference |

* sub-optimal response = any new relapse OR new lesion OR EDSS increase during the first year of treatment
