# Supplementary material for: First-year treatment response predicts the following 5-year disease course in patients with relapsing-remitting multiple sclerosis
Source: Neurotherapeutics. 2025 Feb 17;22(2):e00552. doi: 10.1016/j.neurot.2025.e00552 (PMC12014414; doi:10.1016/j.neurot.2025.e00552)
Supplement: Multimedia component 4 [file mmc4.docx]

**Table S4.** Risk of disability improvement within 5 years from diagnosis

|  |  | **Univariate, Random effects = country & epoch** | **Multivariate, Random effects = country & epoch** | **Multivariate, Random effects = country, epoch & clinic** |
| --- | --- | --- | --- | --- |
| **Explanatory variable** | **Category** | **Hazard Ratio (95% CI) p-value** | **Hazard Ratio (95% CI) p-value** | **Hazard Ratio (95% CI) p-value** |
| Age at baseline (units=10 years) |  | **0.79 (0.74, 0.84) <0.001** | **0.78 (0.73, 0.84) <0.001** | **0.78 (0.73, 0.84) <0.001** |
| Sex | Female | 1.02 (0.89, 1.17) 0.818 | 1.07 (0.93, 1.23) 0.338 | 1.06 (0.92, 1.22) 0.454 |
|  | Male | Reference | Reference | Reference |
|  | Not recorded | Insufficient events | Insufficient events | Insufficient events |
| Months since first symptoms |  | **0.95 (0.93, 0.97) <0.001** | **0.95 (0.93, 0.97) <0.001** | **0.95 (0.94, 0.98) <0.001** |
| First DMT - high efficacy | Yes | 1.18 (0.97, 1.43) 0.090 | 1.00 (0.82, 1.21) 0.971 | 0.99 (0.81, 1.21) 0.949 |
|  | No | Reference | Reference | Reference |
| Baseline EDSS |  | **1.19 (1.13, 1.25) <0.001** | **1.24 (1.16, 1.31) <0.001** | **1.24 (1.16, 1.31) <0.001** |
| Baseline Pyramidal KFS ≥ 2 - n (%) | <2 | Reference | Reference | Reference |
|  | ≥2 | 1.06 (0.92, 1.21) 0.430 | 0.92 (0.79, 1.07) 0.286 | 0.90 (0.77, 1.06) 0.206 |
|  | No baseline pyramidal KFS | 0.69 (0.55, 0.86) 0.001 | 0.69 (0.55, 0.86) 0.001 | 0.71 (0.55, 0.91) 0.006 |
| Baseline Brain MRI - T1 Gd+ lesions | 0 | Reference | Reference | Reference |
|  | 1+ | 1.16 (0.95, 1.41) 0.147 | 1.06 (0.87, 1.30) 0.550 | 1.04 (0.84, 1.28) 0.732 |
|  | MRI performed, lesions not recorded | 0.90 (0.76, 1.06) 0.202 | 0.74 (0.23, 2.38) 0.611 | 0.94 (0.78, 1.14) 0.521 |
| Baseline Brain MRI - T2 lesions | 0 | Reference | Reference | Reference |
|  | 1-2 | 1.63 (0.48, 5.51) 0.435 | 1.24 (0.37, 4.20) 0.730 | 1.22 (0.34, 4.30) 0.761 |
|  | 3-8 | 1.02 (0.32, 3.28) 0.974 | 0.74 (0.23, 2.38) 0.611 | 0.73 (0.22, 2.44) 0.604 |
|  | 9+ | 1.13 (0.35, 3.62) 0.838 | 0.82 (0.26, 2.64) 0.743 | 0.78 (0.23, 2.60) 0.682 |
|  | MRI performed, lesions not recorded | 1.01 (0.32, 3.22) 0.991 | 0.85 (0.27, 2.72) 0.786 | 0.77 (0.23, 2.55) 0.665 |
| Sub-optimal response* in first year of treatment | Yes | 0.93 (0.81, 1.06) 0.271 | 0.91 (0.79, 1.03) 0.146 | 0.90 (0.79, 1.03) 0.134 |
|  | No | Reference | Reference | Reference |

* sub-optimal response = any new relapse OR new lesion OR EDSS increase during the first year of treatment
