# Supplementary material for: First-year treatment response predicts the following 5-year disease course in patients with relapsing-remitting multiple sclerosis
Source: Neurotherapeutics. 2025 Feb 17;22(2):e00552. doi: 10.1016/j.neurot.2025.e00552 (PMC12014414; doi:10.1016/j.neurot.2025.e00552)
Supplement: Multimedia component 5 [file mmc5.docx]

**Table S5.** Risk of reaching EDSS 3.0 within 5 years from diagnosis

|  |  | **Univariate, Random effects = country & epoch** | **Multivariate, Random effects = country & epoch** | **Multivariate, Random effects = country, epoch & clinic** |
| --- | --- | --- | --- | --- |
| **Explanatory variable** | **Category** | **Hazard Ratio (95% CI) p-value** | **Hazard Ratio (95% CI) p-value** | **Hazard Ratio (95% CI) p-value** |
| Age at baseline (units=10 years) |  | **1.25 (1.16, 1.35) <0.001** | **1.21 (1.12, 1.31) <0.001** | **1.20 (1.11, 1.30) <0.001** |
| Sex | Female | 1.07 (0.91, 1.26) 0.423 | 1.03 (0.88, 1.22) 0.681 | 1.03 (0.88, 1.22) 0.687 |
|  | Male | Reference | Reference | Reference |
|  | Not recorded | Insufficient events | Insufficient events | Insufficient events |
| Months since first symptoms |  | **1.02 (1.00, 1.05) 0.033** | 1.02 (1.00, 1.04) 0.063 | **1.02 (1.00, 1.05) 0.043** |
| First DMT - high efficacy | Yes | 1.03 (0.79, 1.35) 0.803 | 1.01 (0.78, 1.31) 0.959 | 0.99 (0.76, 1.30) 0.966 |
|  | No | Reference | Reference | Reference |
| Baseline EDSS |  | **1.86 (1.68, 2.06) <0.001** | **1.93 (1.72, 2.16) <0.001** | **1.96 (1.75, 2.20) <0.001** |
| Baseline Pyramidal KFS ≥ 2 - n (%) | <2 | Reference | Reference | Reference |
|  | ≥2 | **1.97 (1.60, 2.41) <0.001** | 1.10 (0.88, 1.38) 0.392 | 1.18 (0.93, 1.48) 0.168 |
|  | No baseline pyramidal KFS | 1.06 (0.86, 1.31) 0.564 | 0.96 (0.78, 1.19) 0.734 | 1.01 (0.80, 1.28) 0.904 |
| Baseline Brain MRI - T1 Gd+ lesions | 0 | Reference | Reference | Reference |
|  | 1+ | 0.98 (0.76, 1.23) 0.840 | 0.97 (0.77, 1.22) 0.804 | 0.95 (0.75, 1.22) 0.696 |
|  | MRI performed, lesions not recorded | 1.02 (0.85, 1.22) 0.871 | 1.04 (0.85, 1.26) 0.703 | 1.07 (0.87, 1.32) 0.531 |
| Baseline Brain MRI - T2 lesions | 0 | Reference | Reference | Reference |
|  | 1-2 | 0.77 (0.22, 2.68) 0.681 | 0.80 (0.23, 2.80) 0.731 | 0.75 (0.21, 2.69) 0.662 |
|  | 3-8 | 0.83 (0.26, 2.65) 0.754 | 0.92 (0.29, 2.93) 0.884 | 0.91 (0.28, 2.99) 0.875 |
|  | 9+ | 0.78 (0.24, 2.47) 0.668 | 0.77 (0.24, 2.46) 0.662 | 0.80 (0.25, 2.63) 0.719 |
|  | MRI performed, lesions not recorded | 0.76 (0.24, 2.47) 0.633 | 0.81 (0.26, 2.56) 0.718 | 0.87 (0.27, 2.84) 0.822 |
| Sub-optimal response^*^ in first year of treatment | Yes | **2.62 (2.26, 3.04) <0.001** | **2.99 (2.58, 3.47) <0.001** | **3.01 (2.58, 3.51) <0.001** |
|  | No | Reference | Reference | Reference |

* sub-optimal response = any new relapse OR new lesion OR EDSS increase during the first year of treatment
