# Supplementary material for: First-year treatment response predicts the following 5-year disease course in patients with relapsing-remitting multiple sclerosis
Source: Neurotherapeutics. 2025 Feb 17;22(2):e00552. doi: 10.1016/j.neurot.2025.e00552 (PMC12014414; doi:10.1016/j.neurot.2025.e00552)
Supplement: Multimedia component 6 [file mmc6.docx]

**Table S6.** Risk of reaching EDSS 6.0 within 5 years from diagnosis

|  |  | **Univariate, Random effects = country & epoch** | **Multivariate, Random effects = country & epoch** | **Multivariate, Random effects = country, epoch & clinic** |
| --- | --- | --- | --- | --- |
| **Explanatory variable** | **Category** | **Hazard Ratio (95% CI) p-value** | **Hazard Ratio (95% CI) p-value** | **Hazard Ratio (95% CI) p-value** |
| Age at baseline (units=10 years) |  | **1.41 (1.27, 1.56) <0.001** | **1.30 (1.17, 1.44) <0.001** | **1.30 (1.17, 1.45) <0.001** |
| Sex | Female | 0.92 (0.73, 1.15) 0.446 | 0.95 (0.76, 1.19) 0.641 | 0.95 (0.76, 1.20) 0.681 |
|  | Male | Reference | Reference | Reference |
|  | Not recorded | Insufficient events | Insufficient events | Insufficient events |
| Months since first symptoms |  | 1.02 (0.99, 1.05) 0.135 | **1.03 (1.00, 1.07) 0.029** | **1.03 (1.00, 1.07) 0.041** |
| First DMT - high efficacy | Yes | **1.68 (1.20, 2.33) 0.002** | 1.12 (0.80, 1.57) 0.520 | 1.13 (0.80, 1.60) 0.484 |
|  | No | Reference | Reference | Reference |
| Baseline EDSS |  | **1.70 (1.57, 1.84) <0.001** | **1.60 (1.45, 1.76) <0.001** | **1.61 (1.46, 1.78) <0.001** |
| Baseline Pyramidal KFS ≥ 2 - n (%) | <2 | Reference | Reference | Reference |
|  | ≥2 | **2.99 (2.36, 3.78) <0.001** | **1.37 (1.04, 1.81) 0.025** | **1.39 (1.05, 1.83) 0.023** |
|  | No baseline pyramidal KFS | **1.63 (1.22, 2.19) 0.001** | 1.28 (0.94, 1.74) 0.117 | 1.32 (0.80, 1.60) 0.484 |
| Baseline Brain MRI - T1 Gd+ lesions | 0 | Reference | Reference | Reference |
|  | 1+ | 1.05 (0.76, 1.46) 0.757 | 1.11 (0.80, 1.55) 0.526 | 1.13 (0.81, 1.59) 0.470 |
|  | MRI performed, lesions not recorded | 1.05 (0.81, 1.36) 0.696 | 0.97 (0.73, 1.28) 0.819 | 1.00 (0.74, 1.34) 0.992 |
| Baseline Brain MRI - T2 lesions | 0 | Reference | Reference | Reference |
|  | 1-2 | 0.72 (0.17, 2.96) 0.649 | 1.15 (0.28, 4.67) 0.849 | 1.18 (0.28, 4.89) 0.823 |
|  | 3-8 | 0.68 (0.21, 2.23) 0.525 | 1.03 (0.32, 3.35) 0.957 | 1.10 (0.33, 3.65) 0.882 |
|  | 9+ | 0.58 (0.18, 1.90) 0.369 | 0.86 (0.27, 2.77) 0.798 | 0.92 (0.28, 3.05) 0.889 |
|  | MRI performed, lesions not recorded | 0.68 (0.21, 2.19) 0.513 | 1.06 (0.34, 3.38) 0.916 | 1.17 (0.36, 3.83) 0.794 |
| Sub-optimal response^*^ in first year of treatment | Yes | **1.57 (1.27, 1.93) <0.001** | **1.76 (1.43, 2.17) <0.001** | **1.77 (1.43, 2.20) <0.001** |
|  | No | Reference | Reference | Reference |

* sub-optimal response = any new relapse OR new lesion OR EDSS increase during the first year of treatment
