# Supplementary material for: First-year treatment response predicts the following 5-year disease course in patients with relapsing-remitting multiple sclerosis
Source: Neurotherapeutics. 2025 Feb 17;22(2):e00552. doi: 10.1016/j.neurot.2025.e00552 (PMC12014414; doi:10.1016/j.neurot.2025.e00552)
Supplement: Multimedia component 7 [file mmc7.docx]

**Table S7.** Risk of conversion to SPMS within 5 years from diagnosis

|  |  | **Univariate, Random effects = country & epoch** | **Multivariate, Random effects = country & epoch** | **Multivariate, Random effects = country, epoch & clinic** |
| --- | --- | --- | --- | --- |
| **Explanatory variable** | **Category** | **Hazard Ratio (95% CI) p-value** | **Hazard Ratio (95% CI) p-value** | **Hazard Ratio (95% CI) p-value** |
| Age at baseline (units=10 years) |  | **2.29 (1.83, 2.85) <0.001** | **2.05 (1.64, 2.55) <0.001** | **1.78 (1.51, 2.10) <0.001** |
| Sex | Female | **0.53 (0.34, 0.84) 0.007** | **0.53 (0.34, 0.84) 0.007** | **0.61 (0.44, 0.85) 0.004** |
|  | Male | Reference | Reference | Reference |
|  | Not recorded | Insufficient events | Insufficient events | Insufficient events |
| Months since first symptoms |  | 1.04 (0.97, 1.11) 0.239 | 1.04 (0.97, 1.11) 0.278 | 1.04 (0.99, 1.09) 0.107 |
| First DMT - high efficacy | Yes | 0.97 (0.43, 2.15) 0.935 | 0.56 (0.26, 1.20) 0.138 | 0.84 (0.46, 1.53) 0.568 |
|  | No | Reference | Reference | Reference |
| Baseline EDSS |  | **1.37 (1.48, 1.89) <0.001** | **1.59 (1.35, 1.86) <0.001** | **1.31 (1.16, 1.48) <0.001** |
| Baseline Pyramidal KFS ≥ 2 - n (%) | <2 | Reference | Reference | Reference |
|  | ≥2 | **3.66 (2.21, 6.07) <0.001** | 1.36 (0.75, 2.47) 0.306 | **1.74 (1.13, 2.68) 0.012** |
|  | No baseline pyramidal KFS | **2.04 (1.09, 3.82) 0.026** | 1.71 (0.88, 3.33) 0.116 | **1.76 (1.10, 2.81) 0.018** |
| Baseline Brain MRI - T1 Gd+ lesions | 0 | Reference | Reference | Reference |
|  | 1+ | 0.86 (0.45, 1.63) 0.642 | 0.99 (0.51, 1.92) 0.979 | 1.18 (0.72, 1.93) 0.507 |
|  | MRI performed, lesions not recorded | 0.61 (0.36, 1.04) 0.069 | **0.55 (0.32, 0.97) 0.038** | 0.81 (0.52, 1.24) 0.331 |
| Baseline Brain MRI - T2 lesions | 0 | Reference | Reference | Reference |
|  | 1-2 | 0.18 (0.01, 2.93) 0.229 | 0.32 (0.02, 5.26) 0.425 | 0.16 (0.01, 1.84) 0.142 |
|  | 3-8 | 0.26 (0.03, 2.03) 0.198 | 0.49(0.06, 3.84) 0.493 | 0.38 (0.08, 1.75) 0.214 |
|  | 9+ | 0.29 (0.04, 2.21) 0.233 | 0.59 (0.08, 4.51) 0.608 | 0.53 (0.12, 2.38) 0.407 |
|  | MRI performed, lesions not recorded | 0.26 (0.04, 1.94) 0.190 | 0.60 (0.08, 4.53) 0.619 | 0.51 (0.12, 2.24) 0.371 |
| Sub-optimal response^*^ in first year of treatment | Yes | **1.68 (1.07, 2.63) 0.25** | **1.85 (1.17, 2.92) 0.008** | 1.20 (0.87, 1.66) 0.258 |
|  | No | Reference | Reference | Reference |

* sub-optimal response = any new relapse OR new lesion OR EDSS increase during the first year of treatment
