# Supplementary material for: First-year treatment response predicts the following 5-year disease course in patients with relapsing-remitting multiple sclerosis
Source: Neurotherapeutics. 2025 Feb 17;22(2):e00552. doi: 10.1016/j.neurot.2025.e00552 (PMC12014414; doi:10.1016/j.neurot.2025.e00552)
Supplement: Multimedia component 8 [file mmc8.docx]

**Table S8.** Risk of developing new lesions at brain MRI within 5 years from diagnosis

|  |  | **Univariate, Random effects = country & epoch** | **Multivariate, Random effects = country & epoch** | **Multivariate, Random effects = country, epoch & clinic** |
| --- | --- | --- | --- | --- |
| **Explanatory variable** | **Category** | **Hazard Ratio (95% CI) p-value** | **Hazard Ratio (95% CI) p-value** | **Hazard Ratio (95% CI) p-value** |
| Age at baseline (units=10 years) |  | **0.78 (0.73, 0.84) <0.001** | **0.79 (0.73, 0.84) <0.001** | **0.75 (0.70, 0.81) <0.001** |
| Sex | Female | 1.00 (0.87, 1.15) 0.999 | 1.02 (0.89, 1.18) 0.733 | 1.02 (0.88, 1.17) 0.836 |
|  | Male | Reference | Reference | Reference |
|  | Not recorded | Insufficent events | Insufficent events | Insufficent events |
| Months since first symptoms |  | 1.00 (0.98, 1.02) 0.931 | 1.00 (0.98, 1.02) 0.821 | 0.99 (0.97, 1.01) 0.477 |
| First DMT - high efficacy | Yes | **0.78 (0.64, 0.96) 0.020** | **0.78 (0.63, 0.96) 0.021** | 0.87 (0.70, 1.09) 0.240 |
|  | No | Reference | Reference | Reference |
| Baseline EDSS |  | 0.98 (0.94, 1.03) 0.437 | 1.02 (0.96, 1.08) 0.526 | 1.03 (0.96, 1.09) 0.427 |
| Baseline Pyramidal KFS ≥ 2 - n (%) | <2 | Reference | Reference | Reference |
|  | ≥2 | 1.01 (0.87, 1.18) 0.881 | 1.04 (0.86, 1.26) 0.711 | 1.01 (0.83, 1.24) 0.885 |
|  | No baseline pyramidal KFS | **0.56 (0.45, 0.69) <0.001** | **0.65 (0.52, 0.81) <0.001** | **0.65 (0.50, 0.85) 0.002** |
| Baseline Brain MRI - T1 Gd+ lesions | 0 | Reference | Reference | Reference |
|  | 1+ | 1.11 (0.92, 1.34) 0.294 | 1.00 (0.82, 1.21) 0.961 | 1.06 (0.86, 1.30) 0.598 |
|  | MRI performed, lesions not recorded | 0.76 (0.65, 0.90) 0.001 | 0.93 (0.78, 1.10) 0.389 | 1.04 (0.86, 1.26) 0.685 |
| Baseline Brain MRI - T2 lesions | 0 | Reference | Reference | Reference |
|  | 1-2 | 2.54 (0.59, 10.84) 0.209 | 2.53 (0.59, 10.87) 0.211 | 2.44 (0.55, 10.75) 0.240 |
|  | 3-8 | 2.69 (0.66, 11.01) 0.168 | 2.82 (0.69, 11.59) 0.150 | 2.36 (0.56, 9.97) 0.242 |
|  | 9+ | 3.06 (0.75, 12.46) 0.119 | 3.16 (0.77, 12.93) 0.109 | 2.71 (0.64, 11.41) 0.174 |
|  | MRI performed, lesions not recorded | 1.54 (0.38, 6.25) 0.550 | 1.74 (0.43, 7.14) 0.440 | 1.91 (0.45, 8.06) 0.376 |
| Sub-optimal response* in first year of treatment | Yes | **2.42 (2.14, 2.75) <0.001** | **2.37 (2.09, 2.69) <0.001** | **2.33 (2.04, 2.66) <0.001** |
|  | No | Reference | Reference | Reference |

* sub-optimal response = any new relapse OR new lesion OR EDSS increase during the first year of treatment
