# Supplementary material for: First-year treatment response predicts the following 5-year disease course in patients with relapsing-remitting multiple sclerosis
Source: Neurotherapeutics. 2025 Feb 17;22(2):e00552. doi: 10.1016/j.neurot.2025.e00552 (PMC12014414; doi:10.1016/j.neurot.2025.e00552)
Supplement: Multimedia component 9 [file mmc9.docx]

**Table S9.** Risk of developing new lesions at spinal MRI within 5 years from diagnosis

|  |  | **Univariate, Random effects = country & epoch** | **Multivariate, Random effects = country & epoch** | **Multivariate, Random effects = country, epoch & clinic** |
| --- | --- | --- | --- | --- |
| **Explanatory variable** | **Category** | **Hazard Ratio (95% CI) p-value** | **Hazard Ratio (95% CI) p-value** | **Hazard Ratio (95% CI) p-value** |
| Age at baseline (units=10 years) |  | **0.89 (0.79, 0.99) 0.048** | 0.89 (0.78, 1.00) 0.051 | **0.83 (0.73, 0.94) 0.004** |
| Sex | Female | 0.99 (0.77, 1.27) 0.960 | 1.00 (0.78, 1.29) 0.983 | 1.00 (0.78, 1.30) 0.974 |
|  | Male | Reference | Reference | Reference |
|  | Not recorded | Insufficent events | Insufficent events | Insufficent events |
| Months since first symptoms |  | 0.99 (0.96, 1.03) 0.630 | 0.99 (0.96, 1.03) 0.642 | 0.99 (0.95, 1.03) 0.534 |
| First DMT - high efficacy | Yes | 0.73 (0.49, 1.07) 0.109 | 0.69 (0.47, 1.03) 0.072 | 0.95 (0.62, 1.44) 0.793 |
|  | No | Reference | Reference | Reference |
| Baseline EDSS |  | 1.02 (0.93, 1.11) 0.723 | 1.00 (0.90, 1.12) 0.936 | 1.00 (0.89, 1.12) 0.986 |
| Baseline Pyramidal KFS ≥ 2 - n (%) | <2 | Reference | Reference | Reference |
|  | ≥2 | 1.21 (0.92, 1.58) 0.165 | 1.25 (0.90, 1.74) 0.191 | 1.25 (0.89, 1.74) 0.197 |
|  | No baseline pyramidal KFS | **0.38 (0.22, 0.65) <0.001** | **0.49 (0.29, 0.84) 0.010** | **0.43 (0.23, 0.79) 0.007** |
| Baseline Brain MRI - T1 Gd+ lesions | 0 | Reference | Reference | Reference |
|  | 1+ | 0.88 (0.62, 1.23) 0.448 | 0.86 (0.61, 1.21) 0.380 | 0.83 (0.58, 1.119) 0.301 |
|  | MRI performed, lesions not recorded | **0.57 (0.42, 0.77) <0.001** | **0.72 (0.52, 0.99) 0.044** | 0.98 (0.69, 1.41) 0.930 |
| Baseline Brain MRI - T2 lesions | 0 | Reference | Reference | Reference |
|  | 1-2 | 2.33 (0.29, 18.60) 0.426 | 2.25 (0.28, 17.99) 0.444 | 2.17 (0.25, 19.01) 0.483 |
|  | 3-8 | 1.13 (0.15, 8.50) 0.904 | 1.10 (0.15, 0.25) 0.926 | 1.06 (0.13, 8.73) 0.953 |
|  | 9+ | 1.34 (0.18, 10.00) 0.773 | 1.34 (0.18, 9.97) 0.774 | 1.13 (0.14, 9.18) 0.910 |
|  | MRI performed, lesions not recorded | 0.60 (0.08, 4.45) 0.614 | 0.72 (0.10, 5.42) 0.753 | 0.91 (0.11, 7.48) 0.931 |
| Sub-optimal response^*^ in first year of treatment | Yes | **1.70 (1.35, 2.13) <0.001** | **1.63 (1.30, 2.06) <0.001** | **1.65 (1.29, 2.09) <0.001** |
|  | No | Reference | Reference | Reference |

* sub-optimal response = any new relapse OR new lesion OR EDSS increase during the first year of treatment
