# Supplementary material for: First-year treatment response predicts the following 5-year disease course in patients with relapsing-remitting multiple sclerosis
Source: Neurotherapeutics. 2025 Feb 17;22(2):e00552. doi: 10.1016/j.neurot.2025.e00552 (PMC12014414; doi:10.1016/j.neurot.2025.e00552)
Supplement: Multimedia component 10 [file mmc10.docx]

**Table S10.** Risk of achieving PIRA within 5 years from diagnosis.

|  |  | **Univariate, Random effects = country & epoch** | **Multivariate, Random effects = country & epoch** | **Multivariate, Random effects = country, epoch & clinic** |
| --- | --- | --- | --- | --- |
| **Explanatory variable** | **Category** | **Hazard Ratio (95% CI) p-value** | **Hazard Ratio (95% CI) p-value** | **Hazard Ratio (95% CI) p-value** |
| Age at baseline (units=10 years) |  | **1.91 (1.62, 2.25) <0.001** | **1.84 (1.56, 2.17) <0.001** | **1.89 (1.59, 2.24) <0.001** |
| Sex | Female | 0.80 (0.56, 1.16) 0.242 |  |  |
|  | Male | Reference |  |  |
|  | Not recorded | Insufficent events |  |  |
| Months since first symptoms |  | **1.08 (1.03, 1.14) 0.001** | **1.07 (1.02, 1.12) 0.007** | **1.07 (1.01, 1.12) 0.012** |
| First DMT - high efficacy | Yes | 1.25 (0.78, 2.00) 0.361 |  |  |
|  | No | Reference |  |  |
| Baseline EDSS |  | **1.17 (1.04, 1.31) 0.010** | **1.12 (1.01, 1.27) 0.039** | 1.07 (0.94, 1.21) 0.304 |
| Baseline Pyramidal KFS ≥ 2 - n (%) | <2 | Reference |  |  |
|  | ≥2 | **1.83 (1.23, 2.73) 0.003** |  |  |
|  | No baseline pyramidal KFS | 1.27 (0.77, 2.09) 0.352 |  |  |
| Baseline Brain MRI - T1 Gd+ lesions | 0 | Reference |  |  |
|  | 1+ | 0.96 (0.51, 1.82) 0.909 |  |  |
|  | MRI performed, lesions not recorded | 1.43 (0.87, 2.36) 0.157 |  |  |
| Baseline Brain MRI - T2 lesions | <9 | Reference |  |  |
|  | 9+ | 3.09 (0.41, 23.27) 0.272 |  |  |
|  | MRI performed, lesions not recorded | 2.72 (0.37, 19.99) 0.325 |  |  |
| Sub-optimal response* in first year of treatment | Yes | 1.05 (0.74, 1.49) 0.779 |  |  |
|  | No | Reference |  |  |

* sub-optimal response = any new relapse OR new lesion OR EDSS increase during the first year of treatment
