# Supplementary material for: First-year treatment response predicts the following 5-year disease course in patients with relapsing-remitting multiple sclerosis
Source: Neurotherapeutics. 2025 Feb 17;22(2):e00552. doi: 10.1016/j.neurot.2025.e00552 (PMC12014414; doi:10.1016/j.neurot.2025.e00552)
Supplement: Multimedia component 11 [file mmc11.docx]

**Table S11.** Risk of relapses within 5 years from diagnosis in the subgroup of patients treated with moderate efficacy oral DMT (n=201)

|  |  | **Univariate^a^**  **Random effects = country & epoch^b^** | **Multivariate**  **Random effects = country & epoch^b^** |
| --- | --- | --- | --- |
| **Explanatory variable** | **Category** | **Relative Risk (95% CI) p-value** | **Relative Risk (95% CI) p-value** |
| Age at baseline (units=10 years) |  | 0.72 (0.60, 0.87) 0.001 | 0.72 (0.59, 0.88) 0.001 |
| Sex | Female | 1.42 (0.81, 2.50) 0.226 | 1.47 (0.64, 3.38) 0.361 |
|  | Male | Reference | Reference |
| Months since first symptoms |  | 0.97 (0.94, 1.01) 0.096 | 0.99 (0.96, 1.01) 0.175 |
| Baseline EDSS |  | 0.97 (0.78, 1.22) 0.806 | 1.03 (0.97, 1.10) 0.311 |
| Baseline Brain MRI - T1 Gd+ lesions | 0 | Reference | Reference |
|  | 1+ | 2.28 (1.49, 3.49) <0.001 | 2.16 (1.08, 4.35) 0.030 |
|  | MRI performed, lesions not recorded | 1.66 (0.58, 4.75) 0.343 | 1.57 (0.43, 5.75) 0.493 |
| Baseline Brain MRI - T2 lesions | 0 | Reference | Reference |
|  | 1-2 | Insufficient sample | Insufficient sample |
|  | 3-8 | 0.72 (0.33, 1.58) 0.412 | 0.71 (0.36, 1.37) 0.303 |
|  | 9+ | 0.77 (0.41, 1.44) 0.407 | 0.71 (0.31, 1.60) 0.403 |
|  | MRI performed, lesions not recorded | 0.69 (0.58, 0.82) <0.001 | 0.70 (0.40, 1.20) 0.191 |

1. negative binomial regression with robust standard errors (offset for post-baseline follow-up years)
2. Multilevel mixed-effects negative binomial regression (random effect = country, epoch as indicated)
