# Supplementary material for: First-year treatment response predicts the following 5-year disease course in patients with relapsing-remitting multiple sclerosis
Source: Neurotherapeutics. 2025 Feb 17;22(2):e00552. doi: 10.1016/j.neurot.2025.e00552 (PMC12014414; doi:10.1016/j.neurot.2025.e00552)
Supplement: Multimedia component 12 [file mmc12.docx]

**Table S12.** Risk of 6-month confirmed disability progression within 5 years from diagnosis in the subgroup of patients treated with moderate efficacy oral DMT (n=201)

|  |  | **Univariate**  **Random effects = country & epoch^a^** | **Multivariate**  **Random effects = country & epoch^a^** |
| --- | --- | --- | --- |
| **Explanatory variable** | **Category** | **Hazard Ratio (95% CI) p-value** | **Hazard ratio (95% CI) p-value** |
| Age at baseline (units=10 years) |  | 1.62 (1.16, 2.26) 0.005 | 1.46 (1.02, 2.08) 0.039 |
| Sex | Female | 2.67 (0.92, 7.72) 0.070 | 1.81 (0.60, 5.41) 0.291 |
|  | Male | Reference | Reference |
| Months since first symptoms |  | 1.15 (1.04, 1.26) 0.007 | 1.11 (1.00, 1.23) 0.052 |
| Baseline EDSS |  | 1.29 (0.94, 1.78) 0.116 | 1.14 (0.79, 1.63) 0.481 |
| Baseline Brain MRI - T1 Gd+ lesions | 0 | Reference | Reference |
|  | 1+ | 0.17 (0.02, 1.52) 0.113 | 0.27 (0.03, 2.52) 0.250 |
|  | MRI performed, lesions not recorded | 0.60 (0.21, 1.75) 0.353 | 0.75 (0.25, 2.26) 0.614 |
| Baseline Brain MRI - T2 lesions | 0 | Reference | Reference |
|  | 1-2 | Insufficient sample | Insufficient sample |
|  | 3-8 | 1.67 (0.19, 14.26) 0.641 | 2.55 (0.29, 22.72) 0.402 |
|  | 9+ | 0.42 (0.04, 4.03) 0.451 | 0.79 (0.08, 7.78) 0.840 |
|  | MRI performed, lesions not recorded | 1.40 (0.19, 10.47) 0.744 | 1.75 (0.23, 13.39) 0.590 |

1. multilevel mixed effects parametric survival model (with Weibull distribution) (random effect = country, epoch as indicated)
