# Supplementary material for: First-year treatment response predicts the following 5-year disease course in patients with relapsing-remitting multiple sclerosis
Source: Neurotherapeutics. 2025 Feb 17;22(2):e00552. doi: 10.1016/j.neurot.2025.e00552 (PMC12014414; doi:10.1016/j.neurot.2025.e00552)
Supplement: Multimedia component 13 [file mmc13.docx]

**Table S13.** Risk of 6-month confirmed disability improvement* within 5 years from diagnosis in the subgroup of patients treated with moderate efficacy oral DMT (n=201)

|  |  | **Univariate**  **Random effects = country & epoch^a^** | **Multivariate**  **Random effects = country & epoch^a^** |
| --- | --- | --- | --- |
| **Explanatory variable** | **Category** | **Hazard Ratio (95% CI) p-value** | **Hazard ratio (95% CI) p-value** |
| Age at baseline (units=10 years) |  | 1.00 (0.79, 1.27) 0.985 | 0.98 (0.76, 1.26) 0.862 |
| Sex | Female | 0.99 (0.51, 1.90) 0.967 | 1.15 (0.56, 2.33) 0.706 |
|  | Male | Reference | Reference |
| Months since first symptoms |  | 0.95 (0.87, 1.04) 0.287 | 0.94 (0.86, 1.03) 0.207 |
| Baseline EDSS |  | 1.16 (0.81, 1.67) 0.410 | 1.25 (0.85, 1.83) 0.255 |
| Baseline Brain MRI - T1 Gd+ lesions | 0 | Reference | Reference |
|  | 1+ | 0.59 (0.19, 1.81) 0.356 | 0.60 (0.18, 1.97) 0.398 |
|  | MRI performed, lesions not recorded | 0.49 (0.23, 1.07) 0.075 | 0.48 (0.21, 1.09) 0.081 |
| Baseline Brain MRI - T2 lesions | 0 | Reference | Reference |
|  | 1-2 | Insufficient sample | Insufficient sample |
|  | 3-8 | 0.74 (0.24, 2.25) 0.592 | 0.80 (0.24, 2.59) 0.704 |
|  | 9+ | 0.56 (0.19, 1.63) 0.285 | 0.57 (0.19, 1.77) 0.335 |
|  | MRI performed, lesions not recorded | 0.57 (0.22, 1.49) 0.250 | 0.61 (0.22, 1.67) 0.336 |

1. multilevel mixed effects parametric survival model (with Weibull distribution) (random effect = country, epoch as indicated)

*Applies only to patients with a baseline EDSS ≥ 2
