# Supplementary material for: First-year treatment response predicts the following 5-year disease course in patients with relapsing-remitting multiple sclerosis
Source: Neurotherapeutics. 2025 Feb 17;22(2):e00552. doi: 10.1016/j.neurot.2025.e00552 (PMC12014414; doi:10.1016/j.neurot.2025.e00552)
Supplement: Multimedia component 14 [file mmc14.docx]

**Table S14.** Risk of developing new lesions at brain MRI within 5 years from diagnosis in the subgroup of patients treated with moderate efficacy oral DMT (n=201)

|  |  | **Univariate**  **Random effects = country & epoch^a^** | **Multivariate**  **Random effects = country & epoch^a^** |
| --- | --- | --- | --- |
| **Explanatory variable** | **Category** | **Hazard Ratio (95% CI) p-value** | **Hazard ratio (95% CI) p-value** |
| Age at baseline (units=10 years) |  | 0.63 (0.48, 0.81) <0.001 | 0.65 (0.49, 0.84) 0.001 |
| Sex | Female | 0.87 (0.52, 1.44) 0.585 | 1.06 (0.61, 1.83) 0.839 |
|  | Male | Reference | Reference |
| Months since first symptoms |  | 0.99 (0.93, 1.07) 0.877 | 1.00 (0.93, 1.07) 0.969 |
| Baseline EDSS |  | 0.84 (0.67, 1.05) 0.122 | 0.86 (0.67, 1.09) 0.219 |
| Baseline Brain MRI - T1 Gd+ lesions | 0 | Reference | Reference |
|  | 1+ | 1.41 (0.55, 3.63) 0.471 | 1.07 (0.39, 2.95) 0.894 |
|  | MRI performed, lesions not recorded | 0.94 (0.41, 2.19) 0.895 | 0.96 (0.40, 2.32) 0.936 |
| Baseline Brain MRI - T2 lesions | 0 | Reference | Reference |
|  | 1-2 | Insufficient sample | Insufficient sample |
|  | 3-8 | 2.11 (0.63, 7.10) 0.226 | 2.25 (0.62, 8.16) 0.218 |
|  | 9+ | 1.86 (0.58, 5.99) 0.299 | 1.81 (0.50, 6.51) 0.365 |
|  | MRI performed, lesions not recorded | 1.01 (0.33, 3.07) 0.982 | 1.05 (0.33, 3.35) 0.935 |

1. multilevel mixed effects parametric survival model (with Weibull distribution) (random effect = country, epoch as indicated)
