# Supplementary material for: First-year treatment response predicts the following 5-year disease course in patients with relapsing-remitting multiple sclerosis
Source: Neurotherapeutics. 2025 Feb 17;22(2):e00552. doi: 10.1016/j.neurot.2025.e00552 (PMC12014414; doi:10.1016/j.neurot.2025.e00552)
Supplement: Multimedia component 15 [file mmc15.docx]

**Table S15.** Risk of developing new lesions at spine MRI within 5 years from diagnosis in the subgroup of patients treated with moderate efficacy oral DMT (n=201)

|  |  | **Univariate**  **Random effects = country & epoch^a^** | **Multivariate**  **Random effects = country & epoch^a^** |
| --- | --- | --- | --- |
| **Explanatory variable** | **Category** | **Hazard Ratio (95% CI) p-value** | **Hazard ratio (95% CI) p-value** |
| Age at baseline (units=10 years) |  | 0.72 (0.47, 1.10) 0.128 | 0.68 (0.45, 1.02) 0.063 |
| Sex | Female | 1.28 (0.52, 3.17) 0.588 | 1.37 (0.54, 3.46) 0.508 |
|  | Male | Reference | Reference |
| Months since first symptoms |  | 0.95 (0.84, 1.08) 0.471 | 0.96 (0.85, 1.09) 0.510 |
| Baseline EDSS |  | 1.07 (0.74, 1.54) 0.722 | 1.19 (0.80, 1.78) 0.389 |
| Baseline Brain MRI - T1 Gd+ lesions | 0 | Reference | Reference |
|  | 1+ | 1.17 (0.28, 4.93) 0.828 | 1.23 (0.27, 5.67) 0.792 |
|  | MRI performed, lesions not recorded | 0.53 (0.15, 1.91) 0.330 | 0.48 (0.13, 1.78) 0.271 |
| Baseline Brain MRI - T2 lesions | 0 | Reference | Reference |
|  | 1-2 | Insufficient sample | Insufficient sample |
|  | 3-8 | 0.56 (0.09, 3.52) 0.533 | 0.45 (0.08, 2.69) 0.382 |
|  | 9+ | 0.44 (0.08, 2.27) 0.327 | 0.33 (0.06, 1.73) 0.188 |
|  | MRI performed, lesions not recorded | 0.38 (0.07, 1.89) 0.235 | 0.38 (0.08, 1.89) 0.239 |

1. multilevel mixed effects parametric survival model (with Weibull distribution) (random effect = country, epoch as indicated)
