# Supplementary material for: First-year treatment response predicts the following 5-year disease course in patients with relapsing-remitting multiple sclerosis
Source: Neurotherapeutics. 2025 Feb 17;22(2):e00552. doi: 10.1016/j.neurot.2025.e00552 (PMC12014414; doi:10.1016/j.neurot.2025.e00552)
Supplement: Multimedia component 16 [file mmc16.docx]

**Table S16.** Risk of achieving PIRA within 5 years from diagnosis in the subgroup of patients treated with moderate efficacy oral DMT (n=201)

|  |  | **Univariate**  **Random effects = country & epoch^a^** | **Multivariate**  **Random effects = country & epoch^a^** |
| --- | --- | --- | --- |
| **Explanatory variable** | **Category** | **Hazard Ratio (95% CI) p-value** | **Hazard ratio (95% CI) p-value** |
| Age at baseline (units=10 years) |  | 2.39 (1.47, 3.87) <0.001 | 2.42 (1.39, 4.20) 0.002 |
| Sex | Female | 2.22 (0.49, 10.14) 0.303 | 1.17 (0.23, 5.88) 0.846 |
|  | Male | Reference | Reference |
| Months since first symptoms |  | 1.16 (1.01, 1.34) 0.042 | 1.13 (0.97, 1.32) 0.104 |
| Baseline EDSS |  | 1.37 (0.85, 2.22) 0.198 | 1.03 (0.60, 1.75) 0.915 |
| Baseline Brain MRI - T1 Gd+ lesions | 0 | Reference | Reference |
|  | 1+ | Insufficient sample | Insufficient sample |
|  | MRI performed, lesions not recorded | Insufficient sample | Insufficient sample |
| Baseline Brain MRI - T2 lesions | 0 | Reference | Reference |
|  | 1-2 | Insufficient sample | Insufficient sample |
|  | 3-8 | Insufficient sample | Insufficient sample |
|  | 9+ | Insufficient sample | Insufficient sample |
|  | MRI performed, lesions not recorded | Insufficient sample | Insufficient sample |

1. multilevel mixed effects parametric survival model (with Weibull distribution) (random effect = country, epoch as indicated)
